# Brain Systems for Probabilistic and Dynamic Prediction: Computational Specificity and Integration

**DOI:** 10.1371/journal.pbio.1001662

**Published:** 2013-09-24

**Authors:** Jill X. O'Reilly, Saad Jbabdi, Matthew F. S. Rushworth, Timothy E. J. Behrens

**Affiliations:** 1 Centre for Functional Magnetic Resonance Imaging of the Brain (FMRIB), Nuffield Department of Clinical Neurosciences, Oxford University, Oxford, United Kingdom; 2 Department of Experimental Psychology, Oxford University, Oxford, United Kingdom; 3 Wellcome Trust Centre for Neuroimaging, University College London, London, United Kingdom; California Institute of Technology, United States of America

## Abstract

A computational approach to functional specialization suggests that brain systems can be characterized in terms of the types of computations they perform, rather than their sensory or behavioral domains. We contrasted the neural systems associated with two computationally distinct forms of predictive model: a reinforcement-learning model of the environment obtained through experience with discrete events, and continuous dynamic forward modeling. By manipulating the precision with which each type of prediction could be used, we caused participants to shift computational strategies within a single spatial prediction task. Hence (using fMRI) we showed that activity in two brain systems (typically associated with reward learning and motor control) could be dissociated in terms of the forms of computations that were performed there, even when both systems were used to make parallel predictions of the same event. A region in parietal cortex, which was sensitive to the divergence between the predictions of the models and anatomically connected to both computational networks, is proposed to mediate integration of the two predictive modes to produce a single behavioral output.

## Abstract 2

Using computational modelling and neuroimaging, two distinct brain systems are shown to use distinct algorithms to make parallel predictions about the environment. The predictions are then optimally combined to control behavior.

## Author Summary

To interact effectively with the environment, brains must predict future events based on past and current experience. Predictions associated with different behavioural domains of the brain are often associated with different algorithmic forms. For example, whereas the motor system makes dynamic moment-by-moment predictions based on physical world models, the reward system is more typically associated with statistical predictions learned over discrete events. However, in perceptually rich natural environments, behaviour is not neatly segmented into tasks like “reward learning” and “motor control.” Instead, many different types of information are available in parallel. The brain must both select behaviourally relevant information and arbitrate between conflicting predictions. To investigate how the brain balances and integrates different types of predictive information, we set up a task in which humans predicted an object's flight trajectory by using one of two strategies: either a statistical model (based on where objects had often landed in the past) or dynamic calculation of the current flight trajectory. Using fMRI, we show that brain activity switches between different regions of the brain, depending on which predictive strategy was used, even though behavioural output remained the same. Furthermore, we found that brain regions involved in selecting actions took into account the predictions from both competing algorithms, weighting each algorithm optimally in terms of the precision with which it could predict the event of interest. Thus, these distinct brain systems compete to control predictive behaviour.

## Introduction

To function effectively in real time, the brain must continually make predictions of sensory events [1]. To do so, it is hypothesised that the brain constructs internal models of the environment that are tuned through a variety of learning algorithms to reflect the state of the external world [1],[2].

Different behavioural scenarios can require predictive models with dramatically different underlying forms. A lizard attempting to catch a fly on its tongue must predict the instantaneous position of the fly based on rapid extrapolation of the current trajectory under Newtonian physics. By contrast a rat choosing which field to forage in might predict the probability of finding food based on a history of discrete learning events (previous forages) with inherent stochasticity (even if the rat knows for definite that there is a 50% chance of finding food in a certain place on any given visit, he can't know in advance whether he will actually find food on and *particular* visit) [3].

From a computational standpoint, functional specialization in the brain may be defined in terms of the *forms* of generative models that can be represented in different brain areas [2],[4]. For example, some brain areas may be specialized for modelling dynamic systems that are continuous over time; others may model the stochastic probabilities of discrete events and still others may be specialized for the categorization of sensory inputs.

It could be argued that the form of the generative model estimated by a brain system determines the types of sensory information it can process and the types of behaviours it is useful for—the lizard and rat in the previous examples would clearly use different internal models for different behavioural goals. However, in a richly structured environment (such as the natural world) it is entirely possible that representations of the environment with different forms are acquired in parallel. In this case we could ask two questions. First, is it possible that predictions of the same event, with different model forms, are represented in parallel in different brain systems? Second, can the brain combine predictions of different forms to control a single behavioural output?

To investigate these questions, we set up a task in which observers could learn in parallel about two different types of structure within a single environment. Participants were asked to extrapolate the flight path of a moving “space invader” and specifically to predict where it would intersect a certain line on the computer screen (its “landing point”). Within this behavioural context, there was structure in both the dynamic behaviour of the space invader and the stochastic distribution of landing points over many trials. Hence participants could utilize two different forms of internal model to make parallel predictions about the same behaviourally relevant event. We labelled these models *statistical* and *dynamic* and defined them as follows (for summary, see Table 1).

**Table 1:** Summary of the characteristics of two classes of predictive model.

	Statistical Endpoints Distribution	Dynamic Forward Model
Type of data	Discrete, iterative	Continuous, dynamic
Typical instantiation	V_t+1_ = V_t_ + αδ	d^2^Θ/dt^2^ = f(t)
Type of uncertainty	Underlying model is probabilistic, estimate is probabilistic	Underlying model is deterministic, estimate is probabilistic
Time period	Historical/prior	Online
Typical behavioural domain	Reward learning	Action planning

The equations are typical instantiations of each model class—for the statistical endpoints distribution model, a temporal difference learning rule in which the value of an item on iteration (V_t+1_) is equal to the value on iteration t, plus some proportion of the prediction error (δ) times learning rate (α). Dynamic forward models would typically be captured by a set of differential equations, where the rate of change of some parameters (Θ), such as position, is a function of time, f(t).

*Statistical* models consist of probability density functions defining the probability of discrete events—for example, the probabilities that actions or stimuli will be associated with rewards [5]–[7] or the probability of sensory events [8]–[10]. The central feature of these *statistical* models is that predictive representations are built up over a history of discrete events, each representing a sample from an unobserved underlying distribution. An important, related feature is that the predictions made by such models can contain an intrinsic element of randomness or *risk* (expected uncertainty [11]–[13]). Because the models only capture the probability of different outcomes, even if the model exactly captures the underlying distribution, the value of the next sample to be drawn cannot be predicted deterministically. Behaviourally, statistical models could be useful for foraging behaviour (in the wild) or gambling tasks (in the lab).

In contrast, *dynamic* models use the current state and rate of change of a dynamical system to extrapolate its future states across time (as in a differential equation). In these *dynamic forward models*, predictions can be computed via explicit reference to known (or pre-learned) environmental dynamics. In contrast to statistical models, given a set of parameters for a dynamic model, the state of the system at each time point predicts its future states deterministically, and continuously over time. Hence the *form* of the model being estimated is deterministic, even if in practice there is uncertainty about the model *state* or parameters that would best fit with observed data. Dynamic models are central to motor control, where real-time representations of the *predicted* results of motor commands bypass delays associated with sensory feedback. Similarly, dynamic forward models are needed to predict future locations of moving objects [14],[15].

Our hypothesis was that the form of generative model estimated for *statistical* and *dynamic* models was so different that different brain systems would be needed, with neural architectures specialized for the different forms of model. This would result in parallel predictions based on two classes of model, even though both models were used to predict the same observation. It has been suggested that the cerebellum, and the motor system more generally, has circuitry suitable for dynamic modelling [16],[17], while the striatum, together with orbitofrontal cortex [18]–[20], has mechanisms suited to the computation of statistical probability density functions over discrete learning events—for example, the representation of temporally discrete prediction errors [6],[7].

To gain experimental control over the computational strategies used by participants (and hence to identify brain systems associated with those computations), our experimental design exploited the Bayesian concept of precision-weighting. Bayesian logic suggests than when two sources of information are available (such as current observations of a trajectory and a statistical distribution over trajectory endpoints acquired through past experience), they should be weighted according to their relative precision. Using this framework we developed a novel experimental design in which external manipulation of the precision with which two computational mechanisms (statistical and dynamic modelling) could cause participants to shift between two computational strategies, within a single task. This allowed us to identify brain systems associated with each computational mechanism as those which were up- or down-regulated on a trial–to-trial basis as each mechanism became more or less behaviourally relevant.

## Results

The results are presented in two parts. First, using behavioural data and modelling, we justify the assumption that human observers shift the relative weight given to statistical and dynamic estimates of the landing point, according to the relative precisions of the two information sources. The relative weighting given to each predictor shifts proportionally to their relative precision, in accordance with Bayesian theory. Second, we show using functional magnetic resonance imaging (fMRI) that as participants shift between strategies, two separate brain networks (the brain's motor and reward-learning systems) up- and down-regulate their activity in accordance with the behavioural relevance (precision) of probabilistic and dynamic information. Hence we identify a dissociation between two neural systems in terms of the form of generative model calculated therein (dynamic modelling versus estimation of environmental statistics), even when both systems are used to make parallel predictions in a single behavioural context.

**Figure 1:**
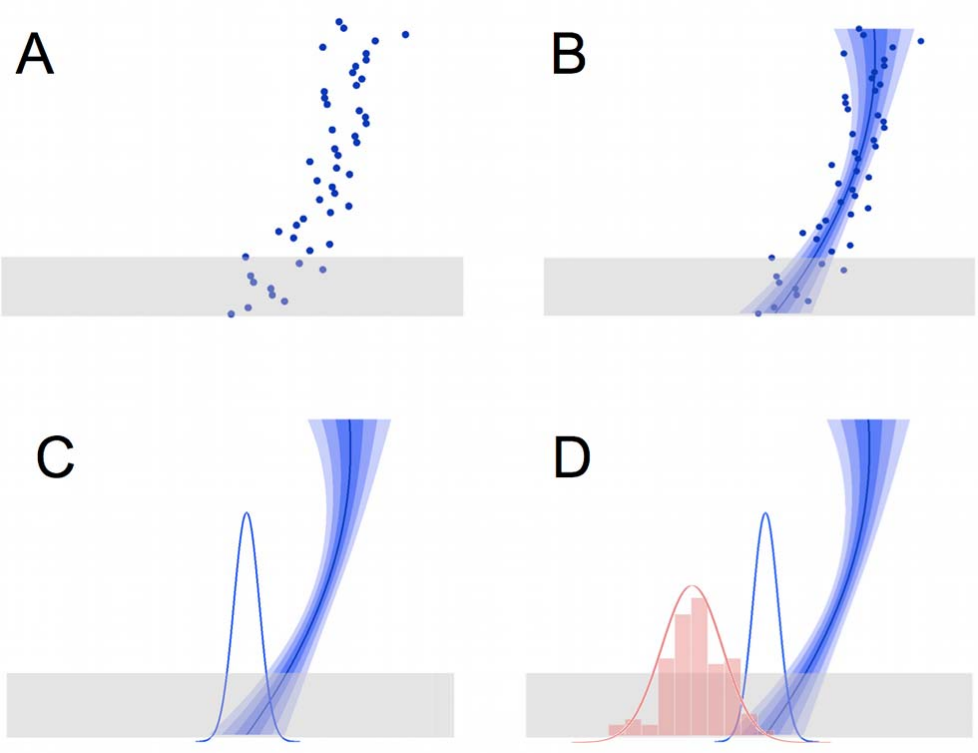
The prediction task. (A) On each trial, participants see a target “space invader” moving down the screen. The target appears at a series of locations in rapid succession (shown here as dots, simultaneously, for illustration) to give the impression of motion. The bottom part of the trajectory is occluded (grey box). Participants must predict where the trajectory will emerge from the occluder (trajectory endpoint). They indicate their response by moving a cursor; after they finalise their response by a button-press, feedback is given, as the target appears at its true endpoint. Trajectories are parabolic, but the start point and curvature are changed randomly on each trial. (B) The participant's estimate of the trajectory was modelled as a quadratic curve. The “best estimate” trajectory is shown here as a solid blue line; the regions indicated by the three levels of blue shading indicate the range of trajectories falling within 1, 2, and 3 standard errors from the best estimated trajectory. (C) This results in a Gaussian probabilistic estimate of the trajectory endpoint (blue bell curve). (D) The trajectory endpoints over many trials (represented by the red histogram) follow a Gaussian distribution (red bell curve), which gives some statistical information a priori about where the endpoint will be. This information can be used to reduce uncertainty in noisy trajectories. The mean and variance of this underlying distribution change periodically and must be learned using a statistical model.

We devised a dynamic modeling task in which participants could use a nondynamic, statistical model of the environment to resolve their uncertainty. Participants had to predict the curved flight trajectory of a “space invader,” judging the horizontal coordinate at which it would intersect a horizontal line near the bottom of the screen (see Figure 1). They observed the first part of the trajectory, but the final section was occluded. To predict where the space invader would “land” at the end of its trajectory (the horizontal coordinate at which it would emerge from the occluded zone), participants had to extrapolate the occluded part of the trajectory. They responded by placing a cursor at the predicted horizontal coordinate of the landing point. The motion of the space invader was governed by a constant acceleration equation in the horizontal dimension, but the acceleration and starting point of the space invader were different on every trial, so in order to predict the trajectory endpoint, participants had to estimate these parameters *jointly* (or equivalently estimate the shape of the emerging trajectory). The fact that both start point and acceleration had to be estimated jointly means that the task was truly a dynamic modeling task in the sense that participants literally had to estimate the entire equation of motion of the space invader, or equivalently a set of differential equations governing its motion, from a series of sequentially presented data points. Estimating the start point or acceleration alone would have been insufficient, because a given start point could result in many possible landing points, if combined with different values of acceleration.

The accuracy with which participants could estimate the equation of motion of the space invader on any given trial was manipulated by adding Gaussian noise to the trajectory—participants were instructed that their “radar equipment” was noisy, but that they should try to guess the underlying trajectory of the space invader as best they could. In noisier trials, trajectory extrapolation was more difficult and hence the precision of the extrapolated trajectory estimate was expected to be lower. In these cases we expected participants to rely more on their a priori estimate of the statistics governing landing points.

Participants were able to use statistical knowledge, because the trajectory endpoints were not uniformly distributed but followed a Gaussian distribution. This statistical model takes the role of a Bayesian prior in our task, because the model consists of a probability density function representing how likely each possible endpoint is a priori, without reference to the actual trajectory observed on the current trial. Note that participants could learn a statistical distribution over the space invaders' landing points entirely independently of their ability to estimate the parameters of the trajectories per se, because they were given feedback about the actual endpoint of each trajectory at the end of each trial. Furthermore, the endpoints were the only environmental statistic that could be predicted. This was the case because both the starting point and curvature of trajectories varied from trial to trial, such that although they *jointly* were selected to give an endpoint following the generative distribution, neither starting point nor curvature *alone* was predictable from trial to trial, or predictive of the current endpoint. Therefore, learning about environmental statistics could be effectively reduced to learning the statistics over endpoints.

**Figure 2:**
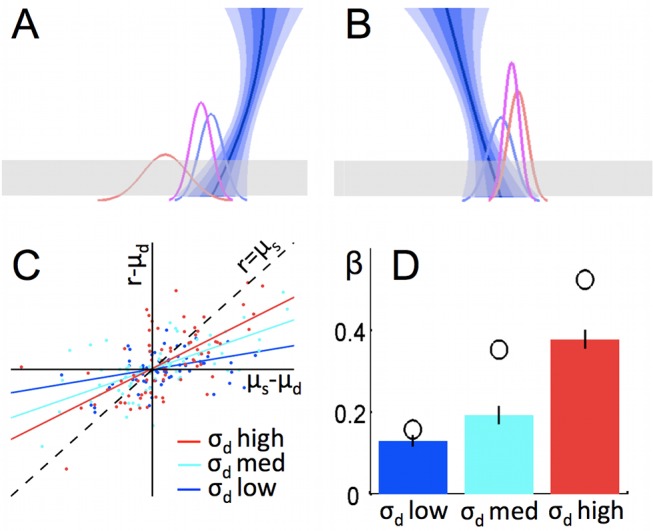
Weighted (Bayesian) combination of predictions. (A and B) On each trial, we hypothesised that participants would make a probabilistic estimate of the trajectory endpoint, using the dynamic forward model (the best estimate of the trajectory and the distribution of possible trajectories is shown by the blue descending line; the corresponding distribution of possible endpoints is shown by the blue Gaussian curve), and that participants have a statistical model of the underlying Gaussian distribution of endpoints over many trials, which is also probabilistic (red Gaussian curve). The optimal way to combine predictions is by precision-weighting (purple). When the trajectory has relatively little noise, (A) the combined estimate of trajectory endpoint is more strongly influenced by the prediction from the dynamic forward model than the statistical model, and vice versa (B). (C) Actual data from a single human participant. Each data point is one trial. On the *x*-axis is displacement of the true trajectory endpoint (*x*) from the mean of the statistical distribution (μ_s_). On the *y*-axis, displacement of the participant's response (r) *from* the true trajectory endpoint (*x*), *towards* the mean of the statistical distribution (μ_s_). If participants relied only on the statistical distribution over many trials, then r would be equal to μ_s_, and hence all points would lie on the line x = y (marked “r = μ_s_”). In contrast, if participants disregarded the statistical model, then responses would simply be centred around the true trajectory endpoint *x*, and hence all points would be distributed about the *x*-axis (*y* = 0). Data points are binned by dynamic model noise level. Responses are more influenced by the statistical distribution (closer to the line x = y and further from the line y = 0) when the observed trajectory is most noisy and therefore the dynamic model is least informative. (D) This effect is significant across the group: bars show mean regression line slope (β) for each bin of trials; error bars are s.e.m. Open circles represent the regression slope for the responses made by the optimal Bayesian observer (weighted combination of predictions with optimal weighting) for the trials in each bin. In a repeated-measures ANOVA for the group of 22 participants, there was a significant linear effect of trajectory noise bin on displacement of the response towards the mean of the statistical distribution (*p* = 0.02). All slopes were significantly above zero (*p*<10^−7^). The slope for σ_d_ med is significantly higher than for σ_d_ low (*p* = 0.0046, paired samples *t* test) and the slope for σ_d_ high is significantly higher than for σ_d_ med (*p*<0.00005, paired samples *t* test).

In order to predict the space invader's landing point on any given trial, the Bayes' optimal solution would be to use all available information, *combining* a dynamic modeling prediction (estimate of the equation of motion) with a prediction from the statistical endpoints distribution, and to weight these strategies according to their relative precisions (Figure 2). We hypothesized that if different brain systems are responsible for the two different types of prediction, activity in each system should be higher when that prediction is behaviorally relevant. Hence we identified areas involved in each strategy as those in which the fMRI signal tracked the relative precision of the prediction from either the dynamic or statistical model.

The relative precision of dynamic and statistical predictions was manipulated on a trial-by-trial basis in two ways:

First, the variance of the Gaussian noise added to each trajectory changed on each trial—making the dynamic prediction more or less precise. We defined trajectory precision (1/σ_d_^2^) as the inverse variance of the estimate of the horizontal landing point, where that estimate was obtained by least-squares fitting of a second order polynomial to the actual observed data points (σ_d_ is the standard error of the estimate of landing point based on the set of observed data points from the current trajectory). This defines an upper bound on the precision with which participants could have predicted the trajectory endpoint from data observed on the current trial alone.

Second, the statistical endpoints' distribution could be made more or less precise because trajectory endpoints were drawn from a series of Gaussian distributions with different variances. The precision of the statistical distribution (1*/σ_s_^2^*) on trial *i* was defined as the inverse variance of the statistical distribution of endpoints that an ideal observer would believe to be in force at the beginning of trial *i*. Again, this defined an upper bound on the precision with which participants could have predicted the landing point, this time using the statistical, nondynamic model.

### 

#### Ideal observer

In the case of the statistical model, it was particularly important to account for participants' incomplete knowledge of the environment, because occasionally (every 20–40 trials), the endpoints' distribution moved to a new position in space or changed its variance. This manipulation, which was introduced to allow us to sample different levels of variance in the underlying distribution and hence the statistical model, meant that participants could never know the true statistics of the environment, but had to learn these over the course of several trials. To account for this incomplete knowledge, we constructed a Bayesian ideal observer model that returned the best estimates of the statistical distribution of trajectory endpoints in force on each trial, given the trials observed so far. Details of the ideal observer model are given in the Methods section and supplementary information (Text S1).

We used an ordinary least squares (OLS) fit of a quadratic curve to generate a prediction based on the trajectory. Although OLS appears rather different from the dynamic processes we are proposing are engaged in the brain, it provides equivalent predictions (about trajectory endpoint) to dynamic estimation of the parameters of a differential equation governing motion. The full dynamic version of the trajectory model and a demonstration of its equivalence in terms of endpoint prediction are presented in Text S1.

To reiterate, throughout the article, all references to parameters of distributions refer to the best estimate an ideal observer could make, given the actual data. These estimated parameters give an upper bound on the accuracy with which participants could perform the task; all modelling of behavioural and fMRI data used these optimal estimates rather than the true parameters of the generative distribution, which only a clairvoyant subject would know. Hence parameters of the statistical model (*μ_s_*, *σ_s_*) used in all equations refer to what our Bayesian ideal observer would believe the statistical distribution to be, and parameters of the dynamic model (*μ_d_*, *σ_d_*) refer to the best least-squares estimate of the current trajectory from the data points actually shown. However, note that where the relative weighting given to the two models was important, we have included free parameters in our models to allow for the possibility that the precision of estimates was suboptimal for one or both types of predictive estimate.

Regarding notation, parameters of the dynamic, statistical, and combined models are denoted by subscripts *d*, *s*, and *sd*, respectively. Throughout the main text, the parameters *μ_s_*, *σ_s_*, *μ_d_*, *σ_d_*, *μ_sd_*, and *σ_sd_* refer to the mean and standard deviation of estimates obtained from the optimal models (the ideal Bayesian observer's estimate of the statistical distribution, the optimal least-squares-fit quadratic trajectory, and the combination of the two as defined in equations 1a–c). These were the parameters used in the modelling of behavioural and fMRI data. The only place in which the true parameters of the generative model are considered (in Text S1) is stated clearly in the text.

### Making a Combined Prediction From the Dynamic and Statistical Models

We hypothesized that participants would use precision-weighting (the Bayes' optimal solution) to combine the two predictive strategies. On each trial, a participant using precision-weighting would combine the prediction from the dynamic forward model with the estimate of the underlying statistical distribution, *weighting* each estimate according to the precision of its prediction (the inverse variance). The consequence of such a *weighted combination model* is that participants' predictions on each trial should not rely solely on data observed on the current trial; rather, predictions should be somewhere between the prediction that would be made based on the current trial alone, and the mean a priori expected endpoint. More weight would be given to the a priori expectations (the statistical model) when the variance of the dynamic trajectory model was high and vice versa (Figure 2a and b).

It was important to verify that participants did indeed use precision weighting, because in the fMRI experiment reported below, we used manipulation of the precision (and hence relevance) of the two information sources to gain experimental control over the relative weighting of the computational models on each trial—hence, the fMRI experiment was premised on the assumption that precision-weighting was used.

Inspection of the data (Figure 2c and 2d) suggests that participants did indeed use precision weighting; grouping trials into bins with high, medium, and low levels of trajectory (dynamic model) noise, the dependence of responses on the statistical model increased significantly as trajectory noise increased (*p* = 0.02, repeated measures ANOVA on regression lines for all participants, for regression of (true trajectory endpoint minus response) on (true trajectory endpoint minus prediction of the statistical model, *μ_s_*)).

**Figure 3:**
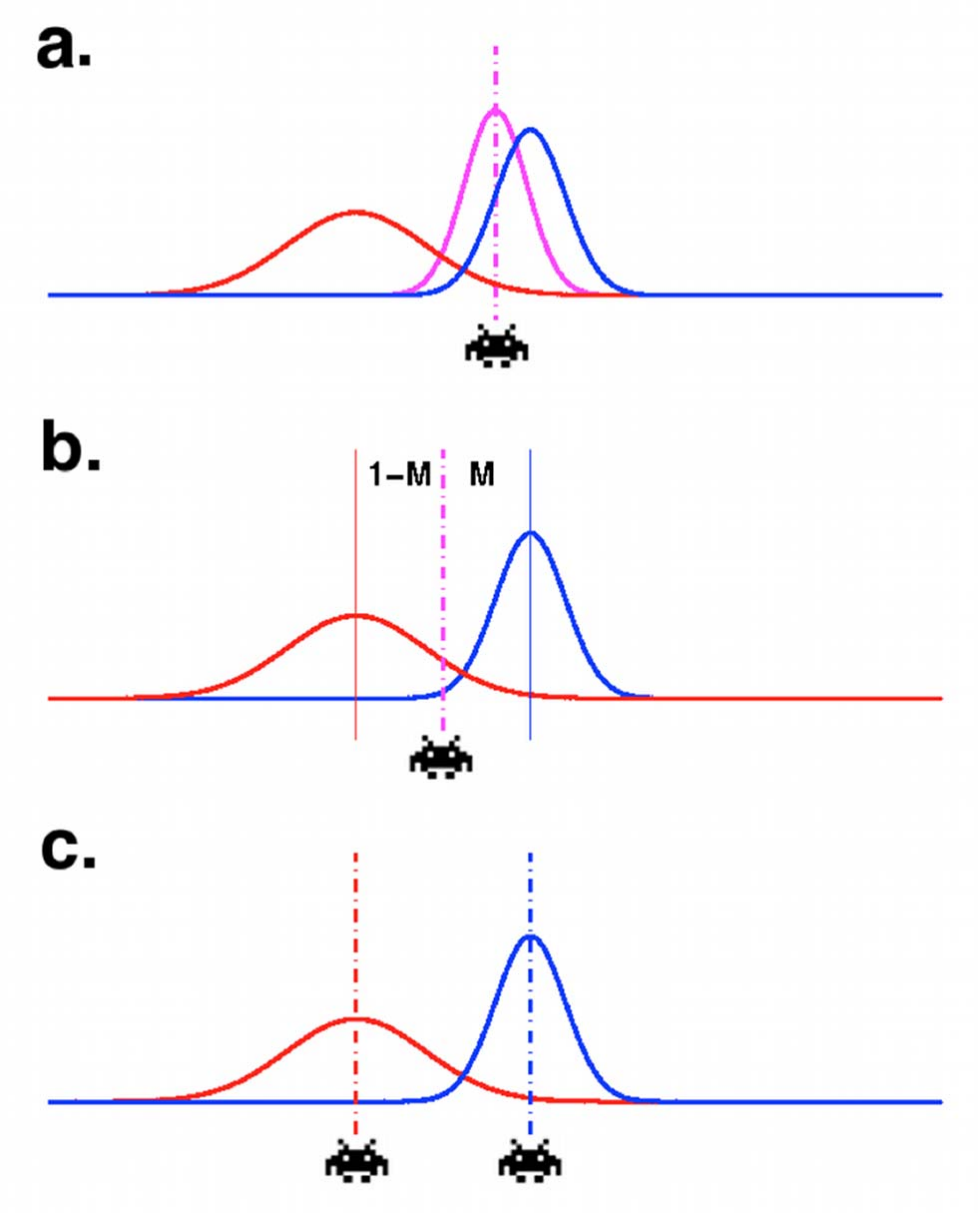
Three alternative ways of combining the trajectory with the statistical model of the environment. Illustration of the three models we compared. In each case a statistical model of the environment over many trials (red) and trajectory estimate (blue) are combined. (a) Weighted combination model—the response is based on the precision-weighted combined distribution (purple). (b) Unweighted combination model—the response in between the predictions from the two models, but does not depend on their relative precision. (c) Weighted noncombination—the actor chooses the prediction with the highest precision but does not combine information from the two predictions.

To test the precision-weighting hypothesis formally, we constructed a *weighted combination* model that generated a set of idealised responses based on a precision-weighting strategy with Gaussian response noise. We compared this model to two alternative models for how the two information sources should be combined: a model that weighted the statistical and dynamic predictions in a fixed ratio, ignoring the fluctuations in their relative precisions (*un-weighted combination model*), and a model that simply chose to act according to the most informative source of information, rather than combining information on each trial (*weighted, no combination model*). The three models are illustrated in Figure 3.

We performed a formal model comparison in which each model was fit to the participants' behavioral data (using individually determined maximum likelihood parameters), and the fits were compared in terms of the model log likelihoods and the Bayesian Information Criterion (BIC).

The three models were defined in terms of how predictions of the statistical model and dynamic model were combined to get a single prediction *μ_sd_*. The three cases can be written as follows, where *μ_s_*, *μ_d_* are, respectively, the mean predictions based on the statistical and dynamic models individually on the trial in question [elsewhere referred to as *μ_s_(i)* etc. for trial *i*, but here we omit the *i* for clarity], *σ^2^_s_* and *σ^2^_d_* are the variance of these estimates, and *b* and *M* are the free parameters: *b* is a constant allowing for spatial bias to the left or right, and *M* is a “mixing factor,” which accounts for any bias to overweight predictions from either the statistical or dynamic model.
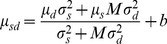
(1a)(weighted combination)
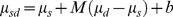
(1b)(un-weighted combination)
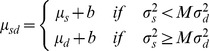
(1c)(weighted, no combination)

We determined model fit on the basis that participants' responses **r** were centred on *μ_sd_* with Gaussian response noise of variance *k^2^*, where *k* was a free parameter in the model: *r∼N(μ_sd_*, *k^2^)*. For each model, for each participant, we found the joint maximum likelihood values for *M*, *k*, and *b* and calculated the model log likelihood as *p(r | r∼N(μ_sd_ , k^2^))*, where *μ_sd_* is determined according to the model equation above, using the MLE values of *b* and *M*.

As they contained the exact same free parameters, the models could be compared according to their log likelihood ratios (logLRs). However, additional nested-model comparison analysis using the Bayesian Information Criterion (see Text S1 and Figure S2) confirms the results. We fit all models to data from 22 participants, who each performed 200 trials of the task while being scanned with fMRI (see below).

The weighted combination model provided the best description of human behavior. It outperformed both the un-weighted combination model (overall logLR = 105, mean ± SEM logLR for individual participants = 4.8±0.83, range = −0.83 to 12.9, logLR >0 for 20/22 participants), and the weighted noncombination model (overall logLR = 363, mean ± SEM for individual participants = 16.5±1.36, range = 7.9 to 30.6, logLR >0 for all participants) (see Figure S1 and Table S1 for full results).

Note that although the alternative combination models are presented here in terms of combining spatial probability density functions over the trajectory endpoint, in fact we *do not* wish to assert that in the brain the two sources of information were combined only at the end of the trajectory or that this combination necessarily occurs in the spatial reference frame of endpoints' coordinates. It seems equally possible that the statistical distribution over *endpoints* acts as a prior over possible *trajectories*, constraining a process of estimating the current trajectory that unfolds as each new data point from the trajectory is observed.

In Text S1, we present a model in which the trajectory is estimated by fitting equations of motion after each observed data point as the trajectory unfolds. In this case, the role of the statistical prior is simply to constrain the set of possible trajectories (Figure S3). In this model, information from the statistical distribution and the current trajectory is truly integrated throughout the trajectory observation process. If information from all data points is linearly combined, this model gives precisely the same predictions of endpoint location (although not necessarily of intermediate locations on the trajectory) as the weighted combination model described above; hence, the behaviour described could certainly be produced by a process in which the statistical model acts as a constraint (a prior) on the trajectory estimation process itself, rather than being combined with the trajectory estimation after the fact. Indeed, since our only measure of behaviour was estimation of the endpoints, we cannot distinguish the two hypotheses.

### Neural Correlates of the Computations

The results of the behavioral modeling indicate that participants did indeed use precision weighting. In accordance with the Bayesian principle that multiple sources of information should be reconciled according to their respective predictive values, participants (a) integrated the output of the two internal models rather than selecting one or the other (weighted combination > weighted noncombination) and (b) weighted the two predictive modes according to their relative precision (weighted combination > un-weighted combination) on a trial-to-trial basis. This finding was the basis for the design of our fMRI experiment.

We used the fact that participants used precision-weighting to shift, parametrically, between strategies as the basis for an fMRI investigation of the neural systems underlying the computations. We reasoned that if the there are computationally specialized neural systems for the two types of prediction, activity in these systems should correlate with how behaviorally relevant that system's prediction was on a trial-to-trial basis. The fact that the weighted combination model fit the behavior of human participants better than the un-weighted combination model indicates that participants made use of each type of predictive computation parametrically, in accordance with its predictive precision. Hence we sought to identify brain regions involved in one or other predictive process as those that track the precision of the prediction for that computational strategy compared to the other, on a trial-to-trial basis.

Causing a trial-to-trial re-weighting of the two modes of information processing is a manipulation analogous to asking participants to attend to one or other aspect of a multidimensional stimulus, in order to up-regulate processing in brain networks involved with that stimulus [21]–[23]. A clear example of this approach is to instruct participants to attend to faces or houses when stimuli are in fact face/house composites [24],[25]. In the present design, manipulations of the relative precision of the two data sources acted essentially as an instruction to alter the relative weighting assigned to the two strategies, and hence to up- or down-regulate brain activity. Therefore, it is the trial-to-trial behavioral relevance of the two types of prediction that we expected to cause a change in the relative activity of brain systems involved in the two types of computation—particularly, we are *not* arguing that manipulating the precision of representations within a brain network causes gross changes in the activity of that network.

We asked two questions about how different computational strategies are implemented in the brain. First, which brain systems are involved in computing each type of prediction (dynamic/statistical)? Second, if there are separate computationally specific neural systems for statistical models versus dynamic models, how is information from these systems integrated in the brain? To address these questions, we used functional magnetic resonance imaging (fMRI). The fMRI results below are from the same 22 participants whose behavioral performance (from the fMRI session) was analyzed above.

We analyzed the fMRI data using a general linear model, with regressors representing the precisions of the dynamic forward model and the statistical model on a trial-to-trial basis (where precision was represented as a parametric modulation of the magnitude of event-related regressors time-locked to the onset of the decision phase of the trial; see Methods). These regressors were log transformed as precision is a logarithm quantity by nature. Formally, the Jeffreys' prior for estimating a precision is uniform if the Bayesian integrals are performed over log precision; intuitively, the same information gain is achieved between precisions of 0.1 and 1, as between precisions of 1 and 10.

A third regressor representing the formation of the combined prediction (the Kullback-Liebler divergence between the statistical model and the combined prediction incorporating dynamic information—see below) was also included. A fourth regressor representing trial-to-trial accuracy (the distance between the participant's prediction and the true landing point) was also used; this regressor was orthogonalized with respect to statistical and dynamic model precision, because as behavioral results show (Figures 2 and 5e) accuracy depended on these variables. All other regressors were uncorrelated (see Methods section for details).

All four regressors were constructed as follows: brain activity was modeled using a single event (short square wave of 0.1 s duration) at the onset of the decision phase of the trial; the magnitude of these events was parametrically modulated to reflect the value of the quantity of interest (e.g., in the case of model precision, larger event magnitudes represented higher precisions). Thus, all regressors had similar temporal/frequency characteristics and represented phasic activity at the time of decision-making, rather than tonic activity over many trials. Therefore, our analysis was sensitive to brain activity associated with representations of the predictive models that were activated at the time or making a decision, rather than with steady-state, stored representations of each model. The four computational regressors were entered into a general linear model together with regressors of no interest representing the main effect of task (events as above, but with an equal magnitude on all trials) and head motion.

The reported group-level statistical maps were thresholded at *p*<0.05 corrected for multiple comparisons at the whole brain level using cluster-based False Discovery Rate correction (see Methods)—this resulted in a minimum cluster size of 238 contiguous 2 mm^3^ voxels at a cluster-forming threshold of *p*<0.01 uncorrected. Additionally, we limit reporting of activations in the main text to only those clusters with a peak Z-score greater than 3.1 (*p*<0.001 uncorrected). However, a full table of activations is given in Table S2.

### Dynamic Forward Modeling and Action Planning

**Figure 4:**
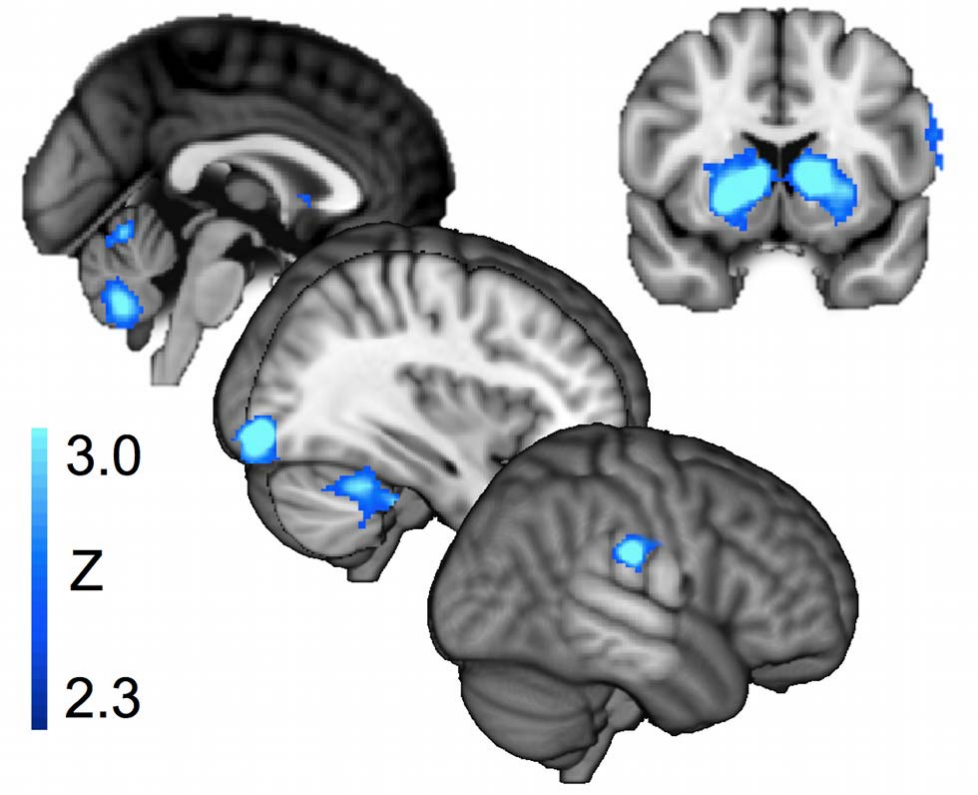
Brain regions associated with the dynamic forward model. Activity correlated with the precision of prediction from the dynamic forward model. Cortical activity and subcortical activity in cerebellum and caudate. The figure shows group Z-maps for the 22 participants, thresholded at *p*<0.05 corrected (see Methods). A full table of activation peaks is given in Table S2.

To extrapolate the occluded part of the trajectory from the observed part, the observer must construct a dynamic forward model representing how the horizontal and vertical position of the target changes over time. Use of the dynamic forward model was correlated with increased activity in a network of connected areas including the anterior inferior parietal cortex in the region of intra-parietal area AIP, the ventral premotor cortex PMv, and connected subcortical areas: Lobules VI and VIII of the cerebellum (AIP is the chief recipient of cerebellar input within IPL and IPS [26]) and the caudate/anterior putamen. These areas are shown in Figure 4 and listed in full in Table S2.

### Statistical Model and Reinforcement Learning

As well as constructing a dynamic forward model of the current trajectory, participants could use a statistical estimate of the underlying distribution to inform their predictions. This estimate of the underlying environmental statistics could be learned by a system without access to the dynamic forward model, because participants were always given feedback on the true endpoint of each trajectory.

**Figure 5:**
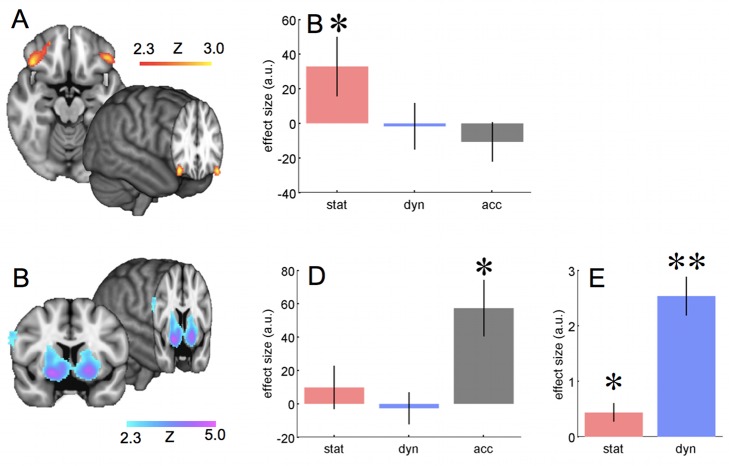
Activity associated with the statistical model and with accuracy. (A) Activity correlated with the precision of prediction from the statistical model. The figure shows group Z-maps for the 22 participants, thresholded at *p*<0.05 corrected (see Methods). (B) Parameter estimates for the effect of precision of the statistical model, precision of the trajectory estimate, and trial-to-trial accuracy, for a region of interest in the orbitofrontal cortex, defined based on a meta-analysis [65]. Bars show group mean, and error bars show s.e.m. Note that although there is a significant effect of precision for the statistical model (*p* = 0.036, one sample *t* test against zero), there is no effect of accuracy per se (*p* = 0.82) or of the precision of the dynamic model (*p* = 0.55); note, in the region of interest analysis, accuracy is not orthogonalised with respect to model precisions, so the effects of model precision are independent of variance that could also be explained by overall accuracy. This is why effect sizes look slightly different to in Figure 6. (C) Activity relating to trial-to-trial accuracy. The figure shows group Z-maps for the 22 participants, thresholded at *p*<0.05 corrected (see Methods). Note the strong peak in the ventral striatum. Slice location is y = 6, peak effect at 20, 6, −10, Z = 4.8. In the whole brain analysis, accuracy was orthogonalised with respect to the model precisions, with which it was correlated (as in panel E). (D) Parameter estimates as in (B), but for a region of interest in the ventral striatum, defined using the nucleus accumbens mask from the Harvard-Oxford atlas, available in FSL (www.fmrib.ox.ac.uk/fsl). Note that this ROI is strongly affected by overall accuracy (*p* = 0.0015, one sample *t* test against zero) but not by the precision of the statistical (*p* = 0.23) or dynamic (*p* = 0.61) models. (E) Behavioral effects of precision of the statistical model and trajectory estimate on accuracy. Bars show group mean ± s.e.m. effect size from a multiple regression of accuracy on precisions for the two models. The effect of precision for both the statistical and dynamic models were significant (*t* test versus zero, *p*<0.01 and *p*<0.0001, respectively), but the effect of dynamic model precision was much greater (paired *t* test, *p*<0.0001).

Activity associated with preferential use of the statistical model was observed in a region more usually associated with reward-learning and calculation of expected value: lateral orbitofrontal cortex (OFC) (Figure 5a, Table S2). This region was activated bilaterally.

### OFC Activity Is Specific to Predictions of the Statistical Model

Since activity in the reinforcement learning system is often associated with predictions of positive outcomes, it could be argued that the activity observed in the OFC and ventral striatum in relation to precision of the statistical model is simply due to an increased expectation of success when endpoints are drawn from a narrow generative distribution. However, increased expectation of success cannot fully explain the current results; instead, it seems that there is an intriguing dissociation between two regions that have both been associated with reward [27]: OFC and the ventral striatum. We contrasted the effects in the two regions of interest of statistical model precision, trajectory model precision, and trial-to-trial accuracy (which in this analysis, unlike the analyses previously presented, was not orthogonalized with respect to the other regressors).

We found that the OFC showed a strong effect of the precision of the statistical model but no effect of trajectory precision or trial-to-trial accuracy, whereas ventral striatum showed a strong effect of accuracy (Figure 5b).

Strikingly, the OFC activity was correlated only with precision of the statistical model, even though the precision of the dynamic trajectory model was a much better predictor of behavioral accuracy than the precision of the statistical model (Figure 5e). These results suggest that whereas OFC may contain a representation of reward expectation [28],[29], this representation is *restricted to the part of the outcome that can be inferred statistically from prior experience*.

An interesting contrast may therefore be drawn between ventral striatal activity, which may be related to success expectation, because it reflects all information bearing on the likelihood of success, and OFC activity, which only reflects that part of the estimate that is furnished by the a statistical model of the underlying environment.

### Combining the Two Models

The results presented so far show that different brain systems are selectively sensitive to predictions based on the dynamic or statistical model. However, behavioral analysis suggested that participants *combine* the predictions of the statistical model and the dynamic trajectory model (see behavioral results). This raises the question: if the statistical model and dynamic trajectory model are combined in the brain, where and how does this happen?

To identify regions in which the statistical and dynamic predictions are combined, two approaches are possible.

First, we might test for regions containing information about both the dynamic and statistical models independently—that is, regions that are independently sensitive to both the precision of the statistical model and the dynamic model. Perhaps surprisingly, when we examined the neural networks associated with precision of the statistical and dynamic models, we found no overlap between the two neural systems (no shared voxels even at a liberal threshold of Z>2.3; that is, *p*<0.01 uncorrected).

Second, we might test for regions that are sensitive to the *disparity* between the predictions of the statistical and dynamic models. In particular, we tested for regions that were active in proportion to how much the prediction of the statistical model had to be updated, with dynamic information, to produce the combined prediction (the Kullback-Liebler divergence between the predictions of the statistical and dynamic model; see Methods). This approach stems from a predictive coding framework [1], in which observations that are well predicted result in lower neural activity than poorly predicted observations [30]–[32]. Hence the response of brain regions combining statistical and dynamic models should be lower if there is high concordance between the two models' predictions, and higher if there is a high disparity between the two models.

**Figure 6:**
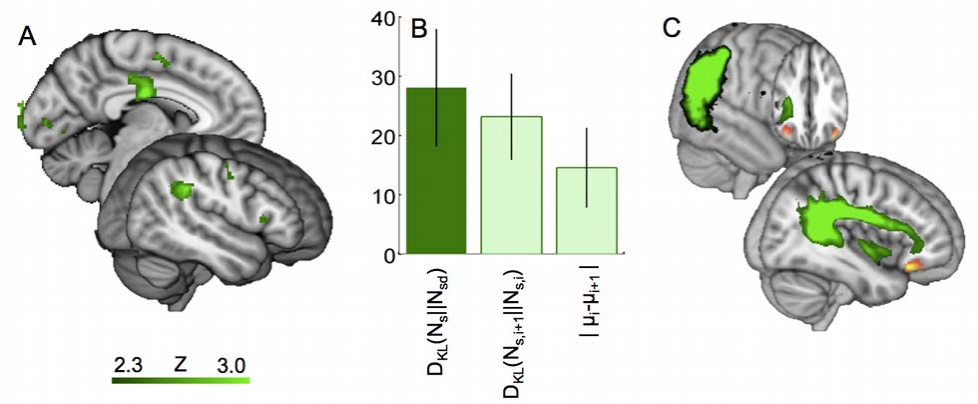
Angular gyrus is a possible site for the formation of the combined prediction. (A) Activity correlated with the degree to which the statistical model must be updated with dynamic information to obtain the combined predictions: 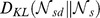 as defined in Equation 3. This statistical map shows results corrected for multiple comparisons using cluster-based correction as described in the Methods section. A full table of activation peaks is given in Table S2. (B) Effects for three measures involving the statistical model, in the angular gyrus. An ROI was defined in the angular gyrus, as all voxels with *p*<0.01 for the effect of divergence between the statistical and combined models, 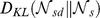 as defined in Equation 3. The group effect is plotted, within this ROI for (i) the degree to which the statistical model must be updated with dynamic information to form the combined prediction 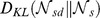
—this was the contrast based on which the ROI was defined so suffers from selection bias—the result is replotted here only for reference; (ii) the degree to which the statistical model is updated between trials, 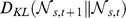 as defined in Equation 4; and (iii) the change in the mean prediction of the statistical model between trials, 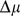 as defined in Equation 5. (C) Diffusion tractography from angular gyrus. Figure shows probabilistic tractography results for a group of 65 subjects, showing the connections of the region in angular gyrus that was active during updating of the statistical model. Connectivity to lateral OFC via the superior longitudinal fasicle can clearly be seen. The image shows, in green, the strength of connectivity of the functionally identified angular gyrus region to each voxel, defined as the average number of “particles” originating in the angular gyrus region which pass through each voxel. The image is thresholded at 1% of total “particles” (minimum) and 10% (maximum). The red region in OFC is the area significantly correlated with the trial-to-trial precision of the statistical model.

We observed activity associated with the disparity between the prediction of the statistical and combined models in the angular gyrus of the IPL, the posterior cingulate cortex, and the putamen (see Table S2 and Figure 6a).

A brain region that forms the combined prediction should have access to the predictions of the statistical model itself. However, none of the regions identified as possible sites of combination were active in proportion to the precision of the statistical model as defined in our analysis above. A possible reason for this would be if the statistical model was coded in the tonic firing, or synaptic efficacy of neurons that are responsive to the trajectory (as we tested only for phasic effects of the precision at the time of decision-making). For example, we might hypothesise that statistical-model-based constraints on the possible sets of motion parameters would be represented by top-down up-regulation of networks of neurons representing the more likely trajectories (we present such a model in Text S1 and Figure S3). In this case, the statistical model could be represented in a region that calculates the dynamic model, but there would not necessarily be a phasic fMRI response correlated with the precision of the statistical model at the time of the decision.

Although a bulk average signal such as BOLD may not be directly sensitive to complex neural coding patterns, it is nevertheless possible to infer the presence of this neural information indirectly, by demonstrating that changes to this complex code are associated with increased BOLD activity. This change-related activity has been used to demonstrate the encoding of specific visual objects [33],[34], actions [35], words [36], and numbers [37] and locations, amongst many other examples.

We therefore tested for representations of the statistical model in each of the possible convergence sites (regions with activity proportional to the KL divergence between statistical and combined predictions) by defining two regressors that captured *changes* in the statistical model from trial to trial: The KL divergence between the predictions of the statistical model on the current and subsequent trials (see Methods) and the simple unsigned change in the mean prediction of the statistical model. Note that because veridical feedback on the landing point was given after each trial, these updates in the statistical model could be, but were not necessarily, calculated without reference to dynamic predictions.

We tested for effects of each of these regressors within the regions in which activity correlated with the prediction error between statistical and combined predictions, identified above: the angular gyrus, putamen, and posterior cingulate. In each case we defined a region of interest as the cluster of voxels with *p*<0.01 uncorrected, identified in the whole brain analysis for the prediction error signal above.

Activity in the angular gyrus was significantly correlated with both these regressors (Figure 6b)—a *t* test for the mean activity in the angular gyrus ROI revealed significant effects of the trial-to-trial KL divergence in the statistical model (*p* = 0.0047) and the change in the mean prediction of the statistical model (*p* = 0.043). These effects were not present in the putamen ROI (*p* = 0.24, *p* = 0.29) or the posterior cingulate ROI (*p* = 0.15, *p* = 0.93).

It is particularly striking that the putative site of convergence in the angular gyrus, which is sensitive to the KL divergence between statistical and combined predictions, is also sensitive to two independently defined regressors that depend on knowledge of the statistical model's predictions (the KL divergence in the statistical model from trial to trial, and the change in its mean prediction), as well as to the disparity (KL divergence), because these latter effects survive even when the regressors are orthogonalized with respect to the KL divergence between the statistical and combined models (*p* = 0.022, *p* = 0.047, respectively—single sample *t* test against zero).

### Angular Gyrus as a Site of Convergence: Connectivity with OFC

It was notable that distinct regions of parietal cortex were associated with trajectory prediction and updating of the statistical model. In particular, the angular gyrus in the posterior inferior parietal lobule (IPL) was active during updating; the angular gyrus is distinct from the more anterior AIP region that we had seen activated in association with trajectory prediction, although the regions are interconnected [38],[39].

Anatomically, the angular gyrus is well placed to provide a bridge for statistical information calculated in frontal striatal systems to reach action maps in parietal cortex. The corresponding region in the macaque, also in the posterior IPL, is distinguished from all other parietal regions by its possession of connections with the lateral OFC [40]; in other words, the same region that we had seen was activated in proportion to the precision of the statistical model (Figure 5a). The connections are reciprocal [40].

Because the connections between posterior IPL (angular gyrus homologue) and lateral OFC are carried in a distinct fascicle in the macaque, the third branch of the superior longitudinal fascicle (SLF III), we were able to use diffusion-weighted imaging and tractography to test for evidence of angular gyrus-lateral OFC connectivity in human subjects. We confirmed that this was the case for the particular region in which activity was associated with the disparity between the statistical and combined predictions by running diffusion tractography on a database of 65 participants, from the region identified in the fMRI experiment above (see Figure 6c).

## Discussion

Traditionally, systems neuroscience has focussed on contrasting different behaviors, tasks, or stimulus types. In contrast, an emerging [2],[4] *computational* view of systems neuroscience suggests that brain systems may be better characterized by the types of internal models they employ, than by the behavioral domains in which they are commonly engaged.

In this study we investigated computational specialization directly by controlling the type of information available to participants, and hence the strategies they could use, in the context of a single goal (predicting the endpoint of a space invader's trajectory). By manipulating their predictive power, we investigated whether different computational strategies for performing the same task recruited different neural systems. Strikingly, we found that each computational domain recruited brain networks that are typically involved in tasks that are computationally similar, but behaviorally dissimilar, to components of the current task. There was preferential involvement of the motoric/action planning regions when participants used a dynamic model to make predictions, and preferential involvement of the reinforcement learning system, particularly lateral OFC, when a statistical model was used. This dissociation occurred even though on all trials participants were making a single behavioural response based on the two predictions.

### Brain Networks with Computational Specificity

In this experiment, we set out to test whether two types of predictive model—dynamic and probabilistic—were associated with different brain systems. We found that there were indeed brain systems computationally specialized for the two types of prediction. These brain systems have generally been associated with different behavioral domains—but those behavioral domains can also be distinguished in terms of the computations involved.

In the current paradigm, dynamic modeling was applied to prediction of a perceptual trajectory, but it was associated with activity in a network of brain areas that have generally been associated with object-directed reaching [41],[42], and with the motor system more generally. Trajectory extrapolation is computationally similar to motor control in that both require a dynamic forward model in which the position of an object (or body part) is represented and extrapolated using an estimate of the rate of displacement. Perceptual trajectory extrapolation has previously been show to activate a similar network to object-directed reaching [14],[15],[43]; the present results suggest that the network might always be active whenever a dynamic forward model is employed.

In contrast, statistical prediction was associated with activity in the lateral OFC, a region more commonly associated with the learning of reward and value [44]. However, the computations involved in reinforcement learning tasks are similar to those involved in learning the statistical model in this task: The statistical model over past endpoints forms a probabilistic prediction for future endpoints, which can be updated via a prediction error when the true landing position is observed. Similar to reward-learning paradigms, learning of the statistical model occurs in discrete steps and the resulting model consists of probabilities assigned to different outcomes.

Interestingly, although in the present study expected value can be predicted by two computational mechanisms, OFC activity is specific to one of them: there is a specific relationship between OFC and the precision of the *statistical model* of the underlying environment. This suggests the hypothesis that the OFC may be specialized to encode expectations about environmental events that are inferred statistically from prior experience, in general—in other words, activity in the OFC may not be indicative of the type of information that is represented (value) but of how the information was inferred (statistically, from discrete events, with an expectation that future events are nondeterministically sampled from that distribution).

Although we have considered our findings in terms of different forms of computations, it is also possible to describe the two sets of activity in terms of the Bayesian concepts of prior and likelihoods. Indeed, it has previously been claimed that prior knowledge might be represented in OFC [45]. However, although in the present study the statistical model plays the role of a Bayesian prior and the dynamic model corresponds to the Bayesian likelihood, it is unlikely that the role each type of prediction plays in Bayesian inference determines which brain structures calculate that model; rather, both priors and likelihoods may be expressed throughout the brain depending on the type of information being modelled. In contrast to the statistical prior expressed in the OFC in the current study, modality-specific priors may be acquired and expressed within modality-specific cortex. For example, the distribution of orientation-tuned receptive field in primary visual cortex reflects the range of distributions experienced in visual development [46],[47], whereas in auditory cortex, categorical activation of neurons reveals priors in the acoustic structure of human speech [48],[49]; auditory priors as expressing in primary auditory cortex can even be rapidly adjusted to maximize the posterior discriminability of behaviourally relevant sounds [50].

### Integrating Predictions

Bayesian theory suggests that when two sources of information are available, they should be combined. Behavioural analysis confirmed that participants did indeed combine statistical and dynamic models in the current task, making use of both on any given trial. We therefore asked where in the brain predictions from two computationally and neurally distinct systems could be combined.

The present results suggest the parietal cortex as a site of integration for the two predictions. A network of areas centered around the IPS was active during trajectory prediction, whereas a specific region in angular gyrus, which has connections to the lateral OFC, was sensitive both to the disparity between statistical and combined predictions, and to updating of the statistical model.

This finding is analogous to previous observations [51],[52] that motoric regions of cortex show reward prediction error signals in tasks in which the level of reward is determined by the choice of action. It illustrates that dynamic predictions made in the motor system can nonetheless be informed by knowledge of the statistical properties of the environment, even if the key brain regions involved in calculating the statistical model lie elsewhere (in the present experiment, use of the statistical model was associated with activity in a region of lateral OFC that has connections to the proposed integration site in the angular gyrus; see Figure 6c).

The parietal cortex is an appealing substrate for integrating predictions because it contains a response-relevant reference frame: IPS is structured as a series of action-centered, spatial representations that might be accessed by environmental statistical models or dynamic forward models (see Culham and Valyear 2006 [53] for review). Notably signals have been observed in the IPS, albeit in separate experiments, which reflect the learning of reward statistics and the forward dynamics of motor control. Neurons in parietal reach region must access dynamic motor predictions as they represent arm position with zero lag feedback [54]. Similarly neurons in the lateral intraparietal area (LIP) update their receptive fields predictively prior to a saccade [42]. However, LIP neurons also reflect statistical prior expectations of the reward value associated with possible saccadic responses [55]–[57]; these expectations are learned over many previous experiences. It is even suggested that different prior experiences may combine in a statistically optimal fashion to predict pre-saccadic LIP firing [58]. Furthermore, IPL has been linked to the updating of internal models even in an abstract, nonmotoric frame of reference [59].

Another situation where it has been clear that the same computation might be performed separately in two different places is in the context of model-based and model-free learning. Indeed, when subjects are performing tasks that can be performed in a model-based or model-free manner, outcome signals in the ventral striatum reflect the integrated prediction of both modelling strategies [60]. Here we have shown how two computations may be combined when a single action is to be selected, and they may do so in cortical regions that prepare the actions themselves.

Because of its topographic mapping and close links to motor output, the IPS has been used extensively as a model system for investigating factors driving behavior. This is of particular importance in single unit studies, which must necessarily focus on a small region of cortex. Cellular activity in the IPS is therefore characterized in exquisite detail in terms of the computational variables found therein. It is unlikely, however, that the IPS is solely itself responsible for the processing underlying these computations. The present results suggest a hypothesis that inputs to IPS could derive from distinct networks, depending on their computational nature.

## Methods

Twenty-two participants (11 females, mean age 28 years, age range 24–35 years) completed the behavioural training and fMRI parts of the experiment. All participants gave informed consent in accordance with the National Health Service Oxfordshire Central Office for Research Ethics Committees (07/Q1603/11).

### Task

The trajectory extrapolation task was as described above: participants observed a noisy trajectory and extrapolated to guess where the endpoint of the trajectory would fall. They moved a cursor to the predicted endpoint by holding down buttons for leftwards or rightwards movement and pressing a third button to finalize their response. After they responded, participants were given feedback as the target reappeared at its true endpoint. The timing of each trial was as follows—duration of the trajectory (40 samples altogether) was 6 s. Participants were able to respond from the moment the space invader disappeared behind the occluder. Feedback was given immediately after the response was made. fMRI responses were modeled based on a single timepoint, the onset of the response period (the point at which the space invader went behind the occluder).

Trajectories were generated as follows: endpoints were selected from a Gaussian distribution (the mean and variance of which changed every 20–40 trials, independently). After the endpoint was selected, a value for the acceleration in *x* (i.e., the curvature of the trajectory) was randomly selected from a uniform distribution that was defined such that the minimum possible acceleration magnitude was zero (straight vertical trajectory) and the maximum acceleration (either leftwards or rightwards) was such that the trajectory encompassed 50% of the horizontal screen width. Trajectories that went off the edge of the screen were discarded.

This method of generating trajectories meant that a given endpoint could be associated with any value of horizontal acceleration and hence, any start point at the top of the screen. Naturally, if any two values out of endpoint, acceleration, and start point were known, the third could be predicted. However, both start point and acceleration would have to be estimated to predict the endpoint, and similarly prior knowledge of the endpoint could only constrain the joint choice of start point and acceleration, not the individual values.

### Protocol

Each participant completed three task phases: first a behavioral training block of 40 trials in which the trajectories had no noise added, so they could learn the general shape of trajectories; second, a further 310 trials of training to familiarize them with the task environment; and third, an fMRI session of 220 trials. The first 20 of these trials had no trajectory noise, to remind participants of the shape of trajectories. These 20 trials were excluded from fMRI analysis.

Participants were not informed that there was a statistical distribution of trajectory endpoints across trials, nor that this distribution changed over time. However, informal debriefing conversations suggested that most participants did in fact notice that there was a statistical pattern to the endpoints, at least some of the time.

Although the precision of the underlying distribution and the trajectory varied independently from trial-to-trial, across the experiment the variance of the endpoints' distribution and the variance of the white noise in the trajectory were of the same order of magnitude—the standard deviation of trajectory data points about the smooth curve of the underlying (generative) trajectory was (averaged across the 200 fMRI trials) 0.67 of the average standard deviation of trajectory endpoints around their generative mean. In modeling the relative weight given to information sources, we took account of possible differences between subjects in the accuracy of estimating each model, by fitting a “weighting factor” to the data (*M* in the equations 1a–c above).

### Bayesian Ideal Observer

Because the distribution of endpoints changed over time, we could not assume that participants knew the true distribution. We therefore constructed a Bayesian computer participant, which learned about the position and variance of the statistical distribution of endpoint from the same information that human participants were given, and we used its “beliefs” to model what participants should know/believe about the endpoints' distribution on a trial-to-trial basis. The Bayesian computer participant is described in detail in Text S1. Here we give a brief summary.

Like all Bayesian models, our computer participant was supplied with a model of the structure of the environment: it “knew” that trajectory endpoints were generated from a Gaussian distribution with unknown mean and variance, and that these parameters could independently jump to totally new values. We did not model how participants would learn these meta-parameters or “rules of the game,” but focused on the period in which they were already well-learned: by the time participants started the fMRI session, they had had an extensive training session (350 trials, 1 hour) to familiarize them with the task environment. We assumed that knowledge of the task structure (distributions were Gaussian, etc.) was transferred from the training to test session, but that estimates of the parameters of the environment (the location and variance of the statistical distribution) were not; this simplifying assumption was introduced as we could not be sure how participants learned in the training session (when they were also learning the structure of the environment) nor how quickly this learning would decay in the several hours/overnight gap between training and test sessions.

The model used an iterative process, which was updated once for each experimental trial, to estimate the values of four free parameters of the endpoints' distribution (free parameters are simply those parameters whose values are estimated from the data): the distribution mean *μ_s_ (i)* on each trial *i*, the standard deviation *σ_s_(i)*, and the independent probabilities α_μ_ and α_σ_ that *μ_s_(i)* and *σ_s_(i)*, respectively, would jump to new values on a given trial. To estimate the parameters, it calculated the likelihood of the current data point given each possible set of parameters *{μ_s_(i), σ_s_(i), α_μ_, α_σ_}*, and inverted this using Bayes' rule to find the likelihood of the *parameters*, given the data point:
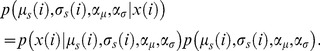
(2)


Initially (before the first trial of the experiment) the model assigned equal probabilities to all values of the parameters, so *p(μ_s_(i), σ_s_(i), α_μ_, α_σ_)* was uniformly distributed across all sets of *{μ_s_(i), σ_s_(i), α_μ_, α_σ_*}_._ After one trial, the probability of each set of parameters *{μ_s_(i), σ_s_(i), α_μ_, α_σ_ }* was updated using Bayes' rule as in equation 1, to give a posterior probability that *{μ_s_(i), σ_s_(i), α_μ_, α_σ_ }* were the true parameters of the distribution. On the next trial, the prior probability of each set of parameters *p(μ_s_(i), σ_s_(i), α_μ_, α_σ_)* was calculated from the posterior of the first trial, using a “leaky” step in which the possibility of a jump in parameter values (as estimated by *α_μ_* and *α_σ_*) was taken into account.

We used the estimates of *μ_s_(i)* and *σ_s_(i)* from the Bayesian computer participant both in the modeling of how people combine the statistical and dynamic models' predictions (above) and in the fMRI analysis.

### fMRI Data Collection and Analysis

In the fMRI block, trial timing was as follows: trajectory observation period lasted 6 s; response was freely timed and took about 1 s on average; and feedback was shown for 500 ms and there was a Poisson-jittered inter trial interval, with a mean ITI of 6 s and the range truncated at 2–12 s.

fMRI data were collected on a Siemens Trio 3 Tesla scanner using an EPI protocol optimized to reduce signal dropout in the inferior frontal cortex [61], with full brain coverage at a resolution of 3×3×3 mm and a temporal resolution of 3.0 s. fMRI data were analyzed using tools from FMRIB software library, FSL [62]: the individual functional images were fieldmap corrected, skull-stripped, and smoothed with a Gaussian kernel at FWHM 8 mm. For group statistics, individual images were registered into MNI space using nonlinear registration tool FNIRT.

fMRI analysis was performed on each individual participant's data using FEAT (from FSL) using a general linear model as described in the main text. The regressors were uncorrelated (correlations: statistical model precision versus dynamic model precision, *r* = −0.0025, *p* = 0.97; statistical model precision versus statistical model update, *r* = 0.11, *p* = 0.10; trajectory model precision versus statistical model update, *r* = 0.040, *p* = 0.57) apart from the regressor representing behavioural accuracy, which was significantly correlated with dynamic model precision (*r* = 0.32, *p*<0.00005). This behavioural accuracy regressor was therefore orthogonalized with respect to other regressors in some analyses as described in the Results section.

At the individual subjects level, regressors in the General Linear Model were defined as the log precision of the statistical and dynamic models, the update of the statistical model (defined as the log KL divergence between the statistical model on the current trial and the next trial as in Equation 4), and the accuracy of the response (the distance in terms of % screen width from the point at which the participant placed the cursor, to the actual endpoint of the underlying trajectory). In some analyses this accuracy regressor was orthogonalized with respect to all other regressors, as stated in the Results section. Contrasts were defined as each regressor minus implicit baseline—that is, the test statistic reported for each regressor is simply the Beta value for that regressor derived from a General Linear Model analysis of the fMRI data.

The measure of disparity between the statistical and combined models was defined as the Kullback Liebler (KL) divergence between the probability distribution of landing points across space based on the statistical model and the distribution based on the combined prediction, incorporating the dynamic model on each trial:
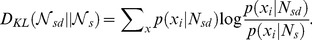
(3)In Equation 3, 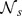 and 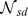 denote the propositions that the landing point on trial *i*, *x_i_*, follows a normal distribution 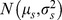 or 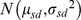 —that is, based on the statistical model alone or on the combined prediction from the statistical and dynamic models, respectively. 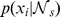 and 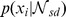 are therefore spatial probability distributions—that is, the probability that each point in space will be the landing point.

The KL divergence between the statistical model on the current and subsequent trials (used to identify steady-state representations of the statistical model) was defined as follows:
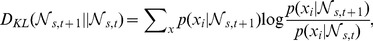
(4)where 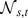 and 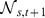 denote the propositions that the landing point on trial *i*, *x_i_*, follows a normal distribution) 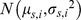 or) 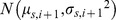
—that is, based on the statistical model at trial *i* or *i+1*, respectively.

The unsigned change in the mean prediction of the statistical model was defined as:
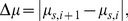
(5)where 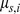 is the value of 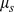 on trial *i*, etc.

Group analysis was done using a random effects model in FEAT. Z (Gaussianised T/F) statistic images were thresholded using clusters determined by voxelwise *p*<0.01 (uncorrected) and a corrected cluster significance threshold of *p* = 0.05 [63]. All statistical maps (figures) and tables show voxelwise statistics (Z-scores), which pass False Discovery Rate correction for multiple comparisons at the whole brain level. Cluster-extent-based false discovery rate was determined using Monte Carlo simulations of a Gaussian random field, matched in size and shape to the MNI brain and with a smoothing kernel of 8 mm (as used in our data analysis), done using the AlphaSim tool distributed with AFNI [64]. Using a cluster forming threshold of Z>2.3 (*p*<0.01), the minimum cluster size necessary to ensure a false discovery rate of α = 0.05 is 238 voxels (in 2 mm MNI space)—hence, only clusters with more than 238 contiguous voxels at this threshold are reported. We imposed an additional criterion of peak Z-score greater than 3.1 (*p*<0.001 uncorrected) for reporting of clusters in the main text, although all clusters surviving the extent threshold above are reported in Table S2.

## Supporting Information

Figure S1 **Model fitting results.** (a) Log likelihood ratio for the two alternative models, the unweighted model and noncombined model, versus the weighted combination model (if the models were equally likely given the data, the logLR would be zero). Participants are ordered according to the log likelihood ratio for weighted versus unweighted model. (b) Maximum likelihood parameters for the three models. The maximum likelihood values for the three free parameters, *M*, *k*, *b* are shown for each model—weighted combination (green), unweighted combination (blue), weighted noncombination (red). Each dot is one participant. Note that *M* had a different range in the three models—in the unweighted combination model *M* ranged from 0 to 1, whereas in the weighted combination and weighted no-combination model, *M* ranged from 0 to 2. For the unweighted combination model (blue dots). 2*M* is plotted so that the whole state space that was searched maps onto the whole plot, for all three models. The purpose of this plot is to illustrate that the state space we searched encompassed the empirical values of *M*, *k*, *b* successfully.
(EPS)

Figure S2 **BIC model comparison.** BIC values for each version of each model in each subject (each version/set of free parameters is one line). Overall the weighted combination model has the best fit for all sets of free parameters in most subjects, whereas there is little difference between different versions of the same model.
(EPS)

Figure S3 **Expanded weighted combination model.** (a) Dynamic model with prior based on the statistical model. A single trial is represented in the whole of panel a; each subplot is a different time-point in the trial. The blue circles represent the actual data points. The blue quadratic curve is the maximum likelihood trajectory given the data points observed so far. The blue Gaussian, superimposed on the trajectory, represents the prediction of where the trajectory will end based on the distribution of possible trajectories, represented as a probability density function over the *x*-coordinate of the endpoint. Notice how the variance decreases as more data points are observed. The red Gaussian is the probability density function for the trajectory endpoint based on the statistical distribution of endpoints. (b) Dynamic model without prior. As above, but in this case the statistical distribution of endpoints (red Gaussian) is shown for reference only—in this version of the model, the statistical distribution is *not* used as a prior. Notice how initially the trajectory estimate is much poorer than the version of the model where the statistical model is used as a prior; the variance decreases as more data points are observed.
(EPS)

Table S1 **Model comparison for behavioural data.** Log Likelihood Ratio (logLR) for each model versus the weighted combination model. Each row is one participant. Participants are ordered by the log likelihood ratio for the weighted combination model versus the model in question, as in the figures. The numbers below the bar are the mean and summed logLR for all participants.
(DOCX)

Table S2 **fMRI results.** Activity relating to (a) precision of the statistical model > precision of dynamic trajectory model, (b) precision of dynamic trajectory model > precision of the statistical model, (c) updating of the statistical model, and (d) trial-to-trial accuracy. Only clusters with a corrected cluster *p* value less than 0.05 are reported.
(DOCX)

Text S1 **Modelling supplement.** A full description of the mathematical models used in the main article.
(PDF)

## Abbreviations

AIP: anterior intra-parietal region
BIC: Bayesian information criterion
BOLD: blood oxygen level dependent signal
fMRI: functional magnetic resonance imaging
IPS: intra parietal sulcus
IPL: inferior parietal lobule
KL Divergence: Kullback-Leibler divergence
LIP: lateral intra-parietal region
OFC: orbitofrontal cortex
OLS: ordinary least squares fit
PMv: ventral premotor cortex
ROI: region of Interest
