# Supplementary material for: Brain Systems for Probabilistic and Dynamic Prediction: Computational Specificity and Integration
Source: PLoS Biol. 2013 Sep 24;11(9):e1001662. doi: 10.1371/journal.pbio.1001662 (PMC3782423; doi:10.1371/journal.pbio.1001662)
Supplement: Table S1 — Model comparison for behavioural data. Log Likelihood Ratio (logLR) for each model versus the weighted combination model. Each row is one participant. Participants are ordered by the log likelihood ratio for the weighted combination model versus the model in question, as in the figures. The numbers below the bar are the mean and summed logLR for all participants. (DOCX) [file pbio.1001662.s004.docx]

| **Weighted, combined model vs. ...** | |
| --- | --- |
| **unweighted** | **uncombined** |
| -0.83 | 13.79 |
| -0.29 | 11.87 |
| 0.60 | 28.37 |
| 0.91 | 11.92 |
| 0.92 | 14.70 |
| 1.80 | 10.84 |
| 1.89 | 19.05 |
| 2.45 | 17.50 |
| 3.01 | 18.15 |
| 3.01 | 15.81 |
| 3.15 | 9.57 |
| 3.38 | 15.13 |
| 4.39 | 9.05 |
| 4.74 | 7.86 |
| 6.46 | 11.58 |
| 7.01 | 17.55 |
| 8.59 | 30.62 |
| 9.43 | 19.28 |
| 9.71 | 20.84 |
| 10.71 | 10.15 |
| 10.94 | 27.06 |
| 12.90 | 22.71 |
|  |  |
| Mean +/- SEM: 4.8+/-0.83 | Mean +/- SEM: 16.50+/-1.36 |
| Total: 105 | Total: 363 |

**Table S1. Model comparison for behavioural data.**

Log Likelihood Ratio (logLR) for each model, vs. the weighted combination model. Each row is one participant. Participants are ordered by the log likelihood ratio for the weighted combination model, vs. the model in question, as in the figures. The numbers below the bar are the mean and summed logLR for all participants.
