# Supplementary material for: Brain Systems for Probabilistic and Dynamic Prediction: Computational Specificity and Integration
Source: PLoS Biol. 2013 Sep 24;11(9):e1001662. doi: 10.1371/journal.pbio.1001662 (PMC3782423; doi:10.1371/journal.pbio.1001662)
Supplement: Table S2 — fMRI results. Activity relating to (a) precision of the statistical model > precision of dynamic trajectory model, (b) precision of dynamic trajectory model > precision of the statistical model, (c) updating of the statistical model, and (d) trial-to-trial accuracy. Only clusters with a corrected cluster p value less than 0.05 are reported. (DOCX) [file pbio.1001662.s005.docx]

|  | Peak voxel location  and Z-score | | | | | Cluster size &  corrected p-value | | | |  |
| --- | --- | --- | --- | --- | --- | --- | --- | --- | --- | --- |
|  | x | y | z | Z | | | k | | p | |
| Precision of statistical model | | | | | |  | |  | |  |
| OFC (L) | 40 | 28 | -18 | | 3.2 | 504 | | 0.003 | |  |
| OFC (R) | -46 | 30 | -20 | | 3.2 | 345 | | 0.007 | |  |
| Precision of dynamic model | | | | | |  | |  | |  |
| AIP (L) | -64 | -26 | 32 | | 3.5 | 481 | | <0.0001 | |  |
| PMv/ BA44 (L) | -60 | 18 | 26 | | 3.2 | 263 | | 0.03 | |  |
| Caudate (L) | -12 | 8 | 6 | | 3.7 | 3015 | | <0.0001 | |  |
| Caudate (R) | 12 | 6 | 2 | | 3.9 |  |  |  |  |  |
| Visual cortex (L) | -30 | -94 | -16 | | 3.6 | 728 | | <0.0001 | |  |
| Visual Cortex (R) | 38 | -92 | -10 | | 4.1 | 672 | | <0.0001 | |  |
| Cerebellum Vermis VIII | 0 | -62 | -38 | | 3.2 | 594 | | <0.0001 | |  |
| Cerebellum Vermis III-IV | 10 | -60 | -12 | | 3.7 |  |  |  | |  |
| Cerebellum R Lobule VI | 36 | -48 | -30 | | 3.0 | 590 | | <0.0001 | |  |
| $D_{KL}(\mathcal{N}_{sd}\vert\left\vert\mathcal{N}_{s} \right)$ | | | | | |  | |  | |  |
| Putamen (R) | 32 | -6 | -6 | | 3.6 | 2168 | | <0.0001 | |  |
| Posterior cingulate | -6 | -16 | 26 | | 3.3 | 1395 | | <0.0001 | |  |
| Angular Gyrus (IPL) | 50 | -36 | 32 | | 3.1 | 472 | | 0.0004 | |  |
| PMv/Area 44 | -46 | 6 | 8 | | 2.7 | 377 | | 0.002 | |  |
| preSMA | 2 | 22 | 48 | | 2.9 | 340 | | 0.004 | |  |
| Frontal pole | -28 | 48 | 12 | | 2.7 | 296 | | 0.01 | |  |
| Anterior Cingulate Sulcus | -14 | 18 | 34 | | 2.8 | 256 | | 0.03 | |  |
| Trial-to-Trial Accuracy | | | | | |  | |  | |  |
| Ventral striatum (R) | 20 | 6 | -10 | | 4.8 | 6726 | | <0.0001 | |  |
| Ventral striatum (L) | -18 | 6 | -6 | | 4.5 |  |  | <0.0001 | |  |
| Visual cortex (L) | -30 | -98 | -6 | | 5.3 | 3166 | | <0.0001 | |  |
| Visual cortex (R) | 36 | -98 | -6 | | 4.0 | 2435 | | <0.0001 | |  |
| Superior frontal gyrus | -16 | 32 | 50 | | 3.9 | 1401 | | <0.0001 | |  |
| IPL (PFt) (L) | -54 | -20 | 45 | | 3.0 | 444 | | 0.0004 | |  |

**Table S2. fMRI results.**

Activity relating to a) precision of the statistical model > precision of dynamic trajectory model b) precision of dynamic trajectory model > precision of the statistical model c) updating of the statistical model d) trial-to-trial accuracy. Only clusters with a corrected cluster p value less than 0.05, are reported. Cluster-size p values were calculated using Monte Carlo simulations implemented with the Alpha Sim tool [[64](#_ENREF_64)]
