# Supplementary material for: Brain Systems for Probabilistic and Dynamic Prediction: Computational Specificity and Integration
Source: PLoS Biol. 2013 Sep 24;11(9):e1001662. doi: 10.1371/journal.pbio.1001662 (PMC3782423; doi:10.1371/journal.pbio.1001662)
Supplement: Text S1 — Modelling supplement. A full description of the mathematical models used in the main article. (PDF) [file pbio.1001662.s006.pdf]

Modelling Supplement

Introduction to modelling section

Throughout the main paper we made use of mathematical models to identify features of interest in both behavioural and fMRI data. In this supplement we describe those models in full.

Before going into details about the models, it is importants to make clear the intended scope of the models presented here. To do this, we draw the reader's attention to the distinction between algorithmic models (the type used in this paper) and mechanistic models. By an algorithmic model we mean a model which describes the computations performed by the brain whilst remaining agnostic about the neural mechanisms which underlie these computations. A mechanistic model or biophysical model might also explain how computations are performed by neurons in terms of action potentials and synaptic transmission; we do not extend the models in this paper to the mechanistic level.

The purpose of algorithmic models, such as those used in this study is to make quantitative predictions about behaviour and brain function by capturing certain aspects of how human participants perform the task. For example, we hypothesised (in accordance with Bayesian logic) that when participants had access to two sources of information about the space invader's trajectory end-point, they should use precision-weighting (see main paper) to combine the sources. To test this hypothesis, we constructed a model that used precision-weighting, and compared it to alternative models which did not use precision-weighting to see which model most closely approximated human participants' behaviour. However, we are *not* suggesting that the equations described below are necessarily the same ones used by the brain to perform the task. In our model, we

worked out the posterior mean and standard deviation using equations for the product of Gaussians, but the brain may arrive at the same posterior distribution using different calculations - for example by simply multiplying probability density functions across a spatial map. The question of interest is only whether the posterior distribution is a *weighted combination* of the two sources, or not. Throughout the modelling work in this paper, we designed models to capture specific aspects of behaviour and/or test specific hypotheses; the models are not supposed to be a complete account of brain function beyond the specific questions they were designed to address.

The models

We constructed our model of the participants' behaviour in several stages.

Firstly and most importantly, the premise of the fMRI experiment was that participants use precision-weighting to decide how much to rely on each of the two computational strategies (statistical and dynamic modelling). To test this hypothesis, we compared predicted endpoints from a precision-weighted model to other non-precision-weighted models.

Secondly, we give a detailed account of how an equation of motion for each observed trajectory could be estimated from the observed data points of that trajectory, on a point-by-point basis, incorporating a prior based on the statistical model of the end-points' distribution over many trials. This model gives behavioural predictions which are precisely equivalent to the precision-weighted model in the model comparison section, because inferences from each observed data point are linearly combined. However, constructing the point-by-point model illustrates how a spatial prior on endpoint (based on the statistical model) can constrain the fitting of a dynamic model of the whole trajectory.

Finally we describe how participants could learn the statistical distribution of end-points. This model is important in that it gives an estimate (or at least, an upper bound) for how well the participants could estimate the end-points' distribution on each trial. Because we needed to sample statistical distributions with different levels of precision, occasionally (every 20-40 trials), the ground-truth statistical end-points' distribution moved to a new position in space or changed its variance. Therefore participants could never know the true statistics of the

environment, but had to learn these from previously observed landing points. To account for subjects' incomplete knowledge of the statistical distribution, we built a Bayesian ideal observer that returned estimates of the current mean and variance of the underlying statistical distribution of end-points, conditional on the landing points witnessed by the participant. Although we present this model last, all references to the statistical model in this section and the main paper refer to what our Bayesian learner would believe the underlying statistical distribution to be, rather than to the actual parameters of the generative distribution which only a clairvoyant subject would know.

All the models described in this supplement were fit using a numerical grid approach in MATLAB. To our knowledge there are no known analytical solutions for the particular models proposed, and furthermore, with the small number of parameters involved, the full posterior could be calculated on a relatively fine grid in just a few minutes on a laptop computer. However, note that although the full posterior was discretized this does not mean that the estimates themselves are discretized, as they are calculated by taking expectations over the posterior.

All the model fits reported in this supplement were conducted only on the data from the fMRI session of the experiment (not the 350 training trials conducted outside the scanner). The first 20 trials of the fMRI session were practice runs in which the trajectory variance was set to zero; these trials were excluded from the model fitting presented here - so all model fits are based on 200 trials from the fMRI session.

1: Do participants use precision-weighting to combine sources of information?

The premise of the fMRI experiment was that participants should shift between two computational strategies (use of the statistical model and use of dynamic trajectory extrapolation) to acheive the same end (i.e. end-point prediction). We hypothesised that as participants adjusted the weighting given to the two strategies, the brain areas involved in each computational system should change their level of activity, upregulating their activity when the computational strat-

egy they computed was more behaviourally relevant and downregulating activity when the laternative strategy was more relevant. Using this logic, we sought to identify brain systems involved in the two computational strategies as those which upregulated their activity when one of the strategies gave a more precise prediction.

To justify this approach in the fMRI analysis it is essential to show that participants do actually adjust how much they rely on each source - historical/statistical and dynamic/trajectory - based on the relative precision of end-point predictions afforded by each model. To pre-empt the results, ultimately precision-weighting did indeed prove to be a good model of how participants actually did the task, and hence we were able to identify brain areas involved in each strategy by looking for fMRI voxels which varied their activity parametrically with the precision of each strategy's prediction.

We performed a formal model comparison to determine whether precision-weighting or some other strategy was used. Let the landing point of the space invader be denoted by x (as only the x-coordinate varied from trial to trial. Then x_s is the landing point predicted by the statistical model alone, x_d is the landing point predicted using the dynamic model from the currently observed trajectory, and x_r is the participant's response. We constructed models in which responses were generated from two Gaussians, the statistical distribution $p(x_s) \sim N(\mu_s, \sigma_s)$, and the distribution of end point estimates given the current observed trajectory, $p(x_d) \sim N(\mu_d, \sigma_d)$. These two distributions were combined in different ways in different models to give a combined prediction x_{sd} with mean μ_{sd}. To evaulate the goodness of fit of each method for generating μ_{sd}, we found the overall model log liklihood for each human participant using the assumption that responses x_r were generated from μ_{sd} (where μ_{sd} was defined based on maximum likelihood values for the free parameters in the model) with Gaussian noise, such that $p(x_r|\mu_{sd}) \sim N(\mu_{sd}, k^2)$. k^2, the response varaince, was fit to the data for each participant. Further details of model evaulation are given below in the 'model comparison' section.

Models for combining the two predictions

We hypothesised that participants would approximate the Bayes'-optimal solution: precision-weighting [1]. In other words, the two sources of information would be combined into a single prediction, taking into account their variance so that the source with the least variance (highest precision) is given more weight. This is a plausible solution because it makes the best use of the available information, and also because it would be the natural result of combining two distributions across a single spatial map - simply by mulitplying the probability given each of two distributions for each point in space.

However, one alternative way in which the two information sources could be used would be to combine them without taking into account their relative precision (e.g. always predict a landing point in half way between the independent predictions for the two models). This could hypothetically occur, for example, if participants didn't have a useful estimate of the precision of each source. If participants used this un-weighted strategy, we would expect both computational brain systems to be equally active on all trials.

A second alternative would be for participants to switch between the two strategies, using only the strategy with the highest precision on any given trial. This might hypothetically occur if the two predictions were made by separate brain systems and for some reason could not be combined. This strategy would imply that the computational systems in the brain should switch between 'on' and 'off' states rather than varying their activity parametrically with precision.

The models are described below, and also summarised in Figure 3 of the main manuscript. Parameters subscripted s refer to the statistical model; parameters subscripted d refer to the dynamic model. Means are labelled μ, standard deviations σ; the free parameters M, k and b are defined below.

1. **Weighted combination.**

$$\mu_{sd} = \frac{1}{\sigma_s^2 + (M\sigma_d)^2}\left(\sigma_s^2\mu_d + (M\sigma_d)^2\mu_s\right) + b \qquad (1)$$

The predicted end-point μ_{sd} is at a position in between the end point predicted by the historical distribution or observed trajectories alone, where the relative

[1]Precision is defined as the inverse of variance

weighting given to each distribution (historical and trajectory) is proportional to its precision.

2. **Non-weighted combination.**

$$\mu_{sd} = \mu_s + M(\mu_s - \mu_d) + b \tag{2}$$

The predicted end point was part way in between the estimates from the two separate predictions; the combined prediction was always a fixed proportion of the way between the two separate predictions, which did not vary from trial to trial and did not depend on the relative precisions of the wo separate predictions.

3. **Weighted, non-combination.**

$$\mu_{sd} = \begin{cases} \mu_s + b & \text{if } \sigma_s^2 < (M\sigma_d)^2 \\ \mu_d + b & \text{otherwise} \end{cases} \tag{3}$$

The predicted end point was identical to *either* the prediction based on the statistical model, or the mean prediction based on the dynamic model, depending which had the highest precision; predictions were not combined within a trial.

Free parameters and input distributions

Models 1-3 each had three free parameters, M, b and k. The joint maximum likelihood (MLE) values for the three parameters (given the actual data) were used; MLE values were selected separately for each model, for each individual.

Mixing factor M. The mixing factor M determined the relative weight given to the dynamic model vs. the statistical model. The reason for doing this was that although we modelled how the variance of each prediction (dynamic and statistical) changed from trial to trial, we did not want to assume that equal values of σ_s and σ_d would be given equal weight; by fitting M we allowed for participants being relatively better at dynamic than statistical modelling and vice versa, and by fitting M for each individual we allowed the relative weight given to each strategy to vary across participants.

Response bias b. We included a bias constant b, which was fit from the data. This allowed a for a tendency to consistently respond to the left or right of the best-fit endpoint and was included on the basis of the informal observation that some participants did indeed tend to show a linear bias in responses.

Response noise k. We modelled participants' reponses as generated from the model prediction μ_{sd} (for each model) with Gaussian noise of unknown variance k^2, where k was fit to the data jointly with M and b.

The factors M, b and in the Bayesian model fitting, the response noise k, were all fit jointly to the data for each individual participant - this process is described in more detail under 'model fitting'. The range for M, k and b was chosen by trial and error to encompass the actual range of MLE values for M, k and b for our participants (see Supplementary Figure 2b). The units of M, k and b are arbitrary, except for in the unweighted combination model, where $0 < M < 1$ (a range which arises from the assumption that the combined estimate always lies between the estimates from the dynamic and statistical models individually).

The units of k and b are correspond to screen pixels; the standard deviation of the statistical distribution, for comparison, was between 20 and 100 pixels. M is a scaling factor with a different meaning in the three models. In the unweighted combination model, $0 < M < 1$; in the other models M could theoretically take any value. **For the weighted combination model, $M/2$ is plotted instead of** M, to match the scale for the other models.

The boundaries of the plot are the boundaries of the state space in which the model worked. These were chosen by trial and error to encompass the actual range of MLE values for M, k and b.

Input distributions. Each model used the same input distributions, which were Gaussian. Let $p(x_s)$ be the probability of any given landing point (in the horizontal x-dimension), given the statistical distribution, and $p(x_d)$ be the probability of a given landing point, given the currently observed trajectory. The statistical distribution $p(x_s) \sim N(\mu_s, \sigma_s)$ was the best estimate of the current underlying distribution of endpoints, based on the joint maximum likelihood values for μ_s, σ_s from the Bayesian learner described in part 3 of this supplement.

The distribution of estimates based on the dynamic model $x_d \sim N(\mu_d, \sigma_d)$ was obtained by fitting a quadratic curve to the observed data points, using least-squares regression; the variance of the trajectory was defined as the variance in the estimate of the x-coordinate of the regression curve, at the point (in y) at which the space invader should 'land'.

Importantly, although for the purposes of model comparison we generated separate predictions of the end point based on the statistical and dynamic models, we do not wish to assert that the brain calculates a dynamic model of the current trajectory without reference to the statistical model, and then combines the two predictions at the end of the trial. On the contrary, in the next section we present a model in which beliefs about the historical distribution influence the estimation of the trajectory as it unfolds, on a point by point basis. However, as will be seen in the next section, the predictions of trajectory end point produced by combining the two models either at the end of the trajectory or throughout its course, are precisely equivalent, because information is combined linearly as each new data point is observed. Since we only have behavioural data for the participants' estimates of trajectory end-point, not intermediate points in the trajectory, the two models are equivalent in terms of predicting human participants' behaviour.

Model comparison

To determine which model gave the best description of the behaviour of human participants, we calculated the log likelihood ratio between models (since all models had the same number of free parameters, there was no need to correct for model complexity).

For each model, we calculated the probability of human participants' responses $\mathbf{x_r}$, given that $\mathbf{x_r} \sim \mathrm{N}(\mu_{\mathbf{sd}}, k^2)$ (where the bold font denotes vector values for $\mathbf{x_r}$ and $\mu_{\mathbf{sd}}$, as these variables take different alues on each trial. Using Bayes' theorem, we calculated the model likelihood for each model, given the data and some set of parameters M, k, b, as $p(\mathbf{x_r} \sim N(\mu_{\mathbf{sd}}, k^2)|\mathbf{x_r}) \propto p(\mathbf{x_r}|\mathbf{x_r} \sim N(\mu_{\mathbf{sd}}, k^2)$. The maximum likelihood values of M, k, b were determined using a numerical grid approach: We calculated model likelihood for a range of combinations of values for $\{M, k, b\}$ and selected the combination which yielded the maximum

model likelihood, given that participant's responses. Following this aproach, the reported model likelihoods are those for the MLE values of M, k, b.

The range of candidate values for M, k, b used in this numerical fitting procedure was chosen by trial and error to encompass the MLE values of all parameters for all participants - MLE values of M, K, b for all subjects are illustrated in Supplementary Figure S1b.

Results

Over the group of subjects, the model which gave the best fit to participants' performance was weighted combination model, followed by the non-weighted combination model (overall logLR for the weighted vs. unweighted model was 105; range in logLR for individual participants was -0.8 to 12.9, mean across participants was 4.8), then the weighted non-combination model (overall logLR for the weighted vs. unweighted model was 363; range in logLR for individual participants was 7.9 to 30.6, mean across participants was 16.5). Log likelihood ratios for the different models are shown in Supplementary Table S1 and Supplementary Figure S1.

BIC analysis

We used the free parameters M and b to fit aspects of the participants behavior that we did not have strong predictions about a-priori (the M parameter was included because we were not sure if some participants would give more weighting to the dynamic of statistical model than might be expected based on optimal precision-weighting) and observations from inspection of the data (b was included as many subjects seemed to show a constant bias to respond to the left- or right- of the optimal endpoint). However, to ensure that the model fit was not affected by the inclusion of unnecessary free parameters, we also calculated the Bayesian Information Criterion (a model fit criterion that penalises models with more free parameters) for versions of each model in which b and M were independently either free or fixed. In summary, all versions of the weighted-combination model out-performed the best-fit version of the unweighted combination model. Lower BIC values indicate a better model fit.

First, we compared versions of each model in which the parameters M and b were either fixed or free. Where b was fixed, its value was set to zero. Where M was fixed, its value was set to reflect optimal performance: in the weighted combination model, M was set to 1, the value it would take if participants used optimal precision weighting; in the non-weighted combination model, M was set to match the ratio of precisions across the whole experiment. This value was obtained using the formula for the mean of the product of two Gaussians:

$$\mu_{sd} = \frac{\sigma_s^2}{\sigma_s^2 + \sigma_d^2}\mu_d + \frac{\sigma_d^2}{\sigma_s^2 + \sigma_d^2}\mu_s \tag{4}$$

hence M_{OPT} was set to the average weighting obtained for the whole set of 200 trials.

$$M_{OPT} = \frac{1}{n}\sum_{1:n}^{t} \frac{\sigma_s^2(t)}{\sigma_s^2(t) + \sigma_d^2(t)} \tag{5}$$

Where t denotes trial number. For the weighted non-combination model M was set to 1.

BIC results

Across participants, there was little difference in BIC scores for the different versions of each model, but all versions of the weighted combination model outperformed all versions of the unweighted combination model and the weighted non-combination model (see Supplementary Figure 2).

We took the best (lowest-BIC) version of each model and directly compared these. The best-fit version of the weighted combination model had no free parameters, and the best fit version of the unweighted combination model also had no free parameters. The difference of BICs was strongly in favour of the weighted combination model (mean+/-SEM difference of BICs=7.9+/-2.1, Range=-18.8-21.8, BIC>0 in 18/22 participants). Difference-of-BIC scores between 6 and 10 are considered 'strong' evidence in favour of the lower-BIC model; difference of BICs > 10 is 'very strong' evidence, after Raftery (1995): Bayesian model selection in social research. Sociological Methodology 25:111-163.

Even when the unweighted combination model was given an unfair advantage y fixing the free parameters at the mean fitted values for the group (mean +/-SEM difference of BICs = 6.4+/-1.6, range=-11.6-18.3, BIC>0 in 18/22 participants), the analysis provided strong evidence that the best-fit version of the weighted combination model was a better fit to data than the unweighted combination model. The weighted non-combination model was a much worse fit than the weighted combination model (mean +/-SEM difference of BICs = 26.8+/12.4, range=5.8-49.5, BIC>0 in 22/22 participants).

Overall the BIC analysis supports our conclusion that the participants used a precision-weighting strategy, but provides little evidence for an effect of the number of free parameters on model fits.

2: How could a spatial prior constrain trajectory extrapolation?

In the previous section we used behavioural evidence to test whether participants used precision-weighting to combine two predictions. In that section we used a simplified model in which the a trajectory of a certain shape (quadratic) was fit to the observed data points, using least squares regression, without reference to the statistical distribution of end points over many trials. This resulted in a probability density function over end-points given the trajectory, which was combined (in different ways for the different models) with a probability density function over end-points given the statistical model, to give a combined prediction. In other words, the dynamic and statistical predictions were calculated separately and combined in the reference frame of the trajectory end-point's coordinates.

However, we *do not* wish to assert that in the brain the two sources of information were combined only at the end of the trajectory, or that this combination necessarily occurs in the spatial reference frame of end-points' coordinates. It seems equally possible that the statistical model acts as a prior over possible trajectories, constraining a process of estimating the current trajectory which unfolds as each new data point from the trajectrory is observed.

The simple model used in the prvious section was useful in that it was com-

putationally undemanding and hence could be run many times to determine the values of free parameters M, k, b. Furthermore, a simple model in which two predictions over endpoint location (from the trajectory and prior) could be used to test different hypotheses about how the predictions were combined (unweighted combination, weighted non-combination, etc).

Conveniently, in terms of endpoint predictions, this simple model is in fact exactly equivalent to a more complex model in which the statistical model constrains the fit of trajectory parameters throughout the trajectory estimation process, which more closely reflects how we hypothesise the brain might combine the two models (which is described in more detail below). The behavioural data we collected only told us where participants thought each trajectory would *end*, and hence the simple model which combines two separate estimates at the end of the trajectory gives exactly the same predictions about behaviour as a model which incorporates the statistical model throught trajectory estimation. Therefore the simplified model was sufficient to test for the use of precision-weighting. However, in this we section present a more complex model to illustrate how a spatial prior can constrain the estimation of a trajectory throughout the observation of that trajectory.

Dynamic integrative model

We hypothesised that people predicted the space invaders' trajectories by constructing a dynamic model of the trajectory which was influenced by prior beliefs about the trajectory's eventual end point based on the statistical model. We define a dynamic model in this case as a model of the velocity and acceleration of the space invader given its position. More generally a dynamic model could be defined as one which describes how the value of a parameter changes over time. A dynamic model could be described mathematically using a set of differential equations.

To model participants' predictions of the space invader's trajectory, we constructed a dynamic model in which the equation of motion of the space invader was estimated, and some sets of parameters were considered a priori more likely because they gave rise to end-points which would be more likely given the statistical model.

To simulate the estimation process in the real task, the model was given each data point from the trajectory sequentially; the model was and updated online as each data point was observed. Essentially the model, which is described in detail below, was a quadratic Kalman filter for the curved trajectory of the space invader.

Generative/'ground truth' form of trajectories

The actual trajectory ('ground truth trajectory') of the space invader on each trial was defined by a constant acceleration equation in the horizontal dimension and a constant velocity in the vertical direction. That is

$$\frac{dy}{dt} = p; \frac{d^2x}{dt^2} = q \tag{6}$$

In fact the velocity in y (the value of p) was fixed across all trials but the acceleration in x (the value of q) varied from trial to trial, as did the trajectory start point x_0. Solving these equations gives a general solution (putting x in terms of y) in the form of a quadratic curve

$$x = ay^2 + by + x_0 \tag{7}$$

In fact, b was always zero (in the ground truth trajectory) but we did not assume participants knew this; we allowed the model to estimate trajectories with different values of b.

So that participants could not predict the trajectory end-point statically from the start point, a new pair of values for a and x_0 were selected on each trial. The trajectories were generated such that their end-points followed a Gaussian distribution (the statistical model which participants estimated) and obviously this constrained the *joint* choice of a and x_0, but neither parameter *individually* could be predicted in advance, or used individually to predict the trajectory's end-point.

The data points presented to the participant were generated from the underlying trajectory by adding Gaussian random noise, in the horizontal dimension, to each observed data point x_i independently. The purpose of including the noise

was to create perceptual uncertainty (participants were told that their “radar signal” was noisy).

Note that although in our model we parameterized the trajectory in terms of x and y rather than x, y and t, there is a unique mapping between each quadratic curve of the form $x = ay^2 + x_0$ and each pair of differential equations, given that p is fixed. In other words, fitting a quadratic curve is simply a re-parameterization and should not be taken to imply that participants estimate a static curve rather than a dynamic equation of motion.

Model

We constructed a Bayesian model that estimated the trajectory online, updating its estimates as each data point from that trajectory was observed. The model assumed that data points were generated following the equation:

$$x_i = ay_i^2 + by_i + x_0 + E_i \tag{8}$$

where E_i is Gaussian noise such that $E_i \sim N(0, \sigma^2)$ and the subscript i denotes observed data points *within* a trajectory.

...in other words, the model ‘knew’ that the space invader had a constant acceleration in x and that noise was Gaussian. The only difference between the form of trajectory estimated by the model and form of the ‘ground truth’ trajectory was that the model did not know that the coefficient of the linear term (b) was always zero.

We used a numerical (grid) method to fit the values of the model’s free parameters after each data point was observed. The model operated on a 4-dimensional state space for a, b, x_0, and σ. After each data point was observed, the probability that each set of parameters a, b, x_0, σ were the correct ones was updated using Bayes’ Rule:

$$p(a, b, x_0, \sigma | x_i) \propto p(x_i | a, b, x_0, \sigma) p(a, b, x_0, \sigma) \tag{9}$$

...where the likelihood $p(x_i | a, b, x_0, \sigma)$ was simply calculated by finding the value of x_i that would be expected given the set of values a, b, x_0 using the

equation:

$$x = ay_i^2 + by_i + x_0 \qquad (10)$$

... and the probability of the observed data point x_i given a, b, x_0 and σ was then calculated using the probability density function for a Gaussian distribution.

The prior $p(a, b, x_0, \sigma)$, for data points $i > 1$, was simply the posterior probability of those parameter values from the previous data point.

For data point $i = 1$, the prior over a, b, x_0, σ was initiated according to participants' prior beliefs about the statistica; distribution of trajectory end points. Trajectory end points followed a Gaussian distribution (see Section 3 below for more details of how this prior was calculated on a trial-to-trial basis). Each set of parameters for the underlying trajectory (a, b, x_0) would predict a certain endpoint $x_{end}|abx_0$. To calculate a prior over the trajectory parameters a, b, x_0 rather than the spatial parameter x_{end}, the probability of the endpoint $x_{end}|abx_0$ given the prior was calculated using the probability density funtion for a Gaussian distribution, and this probability was assigned to the combination of parameters a, b, x_0 as a prior. This was done for each possible combination of a, b, x_0 in turn. Note that the prior was uniform over values for σ because σ was selected randomly on a trial-to-trial basis and did not depend on the hisotical distribution of end-points. Hence there could be no prior expectation about its value at the start of a new trial. The prior over a, b, x_0, σ was normalised and applied in Equation 9 for data point $i = 1$.

Behaviour of the model

To give a flavour of how the model's estimate of the trajectory changes with additional data points, we show the maximum likelihood trajectory at several points during a trial in Supplementary Figure 3. To illustrate how the statistical distribution of end points constrains the trajectory estimate, in Supplementary Figure 3b we show the maximum likelihood trajectory for the same data, but without the prior over a, b, x_0 based on experience of the environmnent's statistics acquired over many trials.

Qualitatively, it can be seen that the greatest difference between models using and ignoring the statistical model actually occurs at the start of the trajectory. When there is relatively little information from the observed trajectory, the prior over endpoints based on the statistical model already constrains the possible trajectory considerably. This is evident from the fact that in the presence of a prior, trajectories ed in roughly the right position even after a few data points, and the variance of the estimated end point (blue Gaussian in the figures) is very much lower (at the start of the trajectory) in the presence of a prior. It is therefore clearly evident how a spatial prior over a trajectory end point can aid estimation of the trajectory when only the very early section of the trajectory is observed.

Equivalence to the model in section 1

We earlier stated without proof that the full model presented here gives a prediction of behaviour which is exactly equivalent to that obtained from the weighted combination model in section 1, if behaviour is constrained to estimating the end point of the trajectory (indeed, this is the only measure we have of participants' trajectory estimates).

This equivalence arises from the multiplicative nature of PDFs. The probability of a landing point x_{end} after observing datapoint n,

$$p(x_{\text{end}}|x_0, x_1, x_2 ... x_n) \tag{11}$$

can be written in terms of the probabilities of x_{end} after each data point:

$$\begin{aligned}
&p(x_{\text{end}}|x_0, x_1, x_2 ... x_n) \\
&= p(x_{\text{end}}|x_0, x_1, x_2 ... x_{n-1})p(x_{\text{end}}|x_n) \\
&= \textstyle\prod_{i=0}^{i} p(x_{\text{end}}|x_i) \\
&= p(x_{\text{end}}|x_0)p(x_{\text{end}}|x_1, x_2 ... x_n) \\
&= p(x_{\text{end}}|\text{historical prior})p(x_{\text{end}}|\text{full trajectory})
\end{aligned} \tag{12}$$

In the full model (this section) we estimate the PDF over x_{end} (or equivalently, over a, b, x_0, σ) sequentially for each data point, using the PDF at data point x_{i-1} as the prior at data point i, and using the statistical model as the prior at data point x_i (or equivalently, as the PDF for an initial 'data point' x_0). But as can be seen from equation 12, this is exactly equivalent to fitting a PDF over x_{end}, or equivalently over a, b, x_0, σ, to all the data points $x_1 \ldots x_p$ simulatneously, and then combining it with the prior based on the statistical model x_i - as we did in the simplified model in section 1.

Modelling participants' estimate of the statistical distribution of end-points

We hypothesised that participants used a priori knowledge about the statistical distribution of trajectory endpoints to resolve uncertainty when the observed trajectory was noisy. However, because the underlying distribution of endpoints (generative distribution) changed periodically, a model in which participants used the true generative endpoints' distribution was not realistic - how could they know what that distribution was on trials when that distribution had just changed? Yet the change in the true prior was an essential part of the design, because we needed to sample distributions with different variances.

To give a more realistic estimate of what participants' statistical model of the end-points' distribution was on any given trial, we created a Bayesian 'computer participant'. This 'computer participant' was a Baysian ideal observer and hence its beliefs about the historical distribution on each trial represented the most accurate estimate an observer could have, given the data points observed.

We did not have direct access to participants' estimates of the historical distribution, because we only measured their predictions in the presence of both the historical distribution and the trajectory. Hence, we could not readily measure whether the Bayesian learner gave a good fit to their actual learning and it can only be said that the present model gives an upper bound on the accuracy and precision of the statistical model, which must be a better estimate of participants' real beliefs than simply using the actual (generative/ ground truth) distribution, which only a clairvoyent subject would know. Note however that

the precise method of learning is not really relevant to the main argument (that participants use precision-weighting) and the purpose of this model is only to improve our estimate of participants' beliefs in order to test whether precision-weighting was used.

Bayesian computer participant

Our Bayesian learner operated as follows: at the start of each trial it had a model (a prior) of the end-points' distribution from its experience of the environmnet's statistics before that trial. When a new end-point was observed, this prior was updated using Bayes' theorem. The Bayesian learner learned entirely based on the true end point on each trial, which was shown to participants at the end of the trial as feedback. Therefore the Bayesian learner could theoretically operate without any access to dynamic estimates of the trajectories' shapes.

Generative model

The Bayesian learner was provided with an accurate model of the shape of the generative distribution of end-points and the fact that its parameters could jump to new values; we simply assumed that participants had learned the overall structure of the environment during the training session of 1 hour / 350 trials. However, the parameters of the distribution and frequency of jumps were modelled as free parameters.

Trajectory end points were drawn from a Gaussian distribution with unknown mean $\mu(t)$ and variance $\sigma^2(t)$; these are the underlying values of which $\mu_s(t)$ and $\sigma_s^2(t)$ are estimators. **For clarity we omit the subscript s in this entire section, and the subscript t now denotes trial number.**

Hence:

$$p(x_t = x|\mu_t, \sigma_t) \sim N(\mu_t, \sigma_t)) \tag{13}$$

...where x denotes the trajectory endpoint, previously called x_{end}

The mean and variance of this Gaussian could 'jump' independently to completely new values. Following a jump, all values of the jumped parameter (mean

or variance) were considered equally likely, so that, if J_μ and J_σ are binary variables representing whether a jump occurred in μ or σ respectively. J_μ and J_σ follow a binomial distributions with probabilites $\alpha_m u$ and $\alpha_s igma$ respectively; these probabilities are free parameters in the model (i.e. they are inferred from the data).

$$\mu_t : \begin{cases} \mu_t = \mu_{t-1} & J_\mu = 0 \\ \mu_t \sim U(\mu_{min}, \mu_{max}) & J_\mu = 1 \end{cases} \tag{14}$$

$$\sigma_t : \begin{cases} \sigma_t = \sigma_{t-1} & J_\sigma = 0 \\ \sigma_t \sim U(\sigma_{min}, \sigma_{max}) & J_\sigma = 1 \end{cases} \tag{15}$$

The probability of a jump in μ or σ on any given trial had a fixed but unknown value $(\alpha_\mu, \alpha_\sigma)$; hence taking together the cases $J = 0, 1$ (i.e. marginalising over J) the conditional priors on values of μ and σ at trial t were:

$$\begin{aligned} p(\mu_t|\mu_{t-1}, \alpha_\mu) = &\quad (1-\alpha_\mu)\mu_{t-1} + \alpha_\mu U(\mu_{min}, \mu_{max}) \\ p(\sigma_t|\sigma_{t-1}, \alpha_\sigma) = &\quad (1-\alpha_\sigma)\sigma_{t-1} + \alpha_\sigma U(\sigma_{min}, \sigma_{max}) \end{aligned} \tag{16}$$

Thus there were four independent free parameters which the Bayesian participant had to estimate from the data at each time point: the mean (μ_t) and variance (σ_t) of the endpoints' distribution, and the independent probabilities (α_μ and α_σ) that each of these parameters would jump on a given trial. These parameters were estimated by inverting the generative model (just described) at each trial using Bayes' rule with the posterior from trial t-1 acting as the prior at trial t. The steps were as follows:

First, to calculate the prior at trial t, compute the joint distribution over $\{\mu_t, \sigma_t, \alpha_\mu, \alpha_\sigma\}$ based on observed data points $x_{1:t-1}$

$$\begin{aligned} &p(\mu_t, \mu_{t-1}, \sigma_t, \sigma_{t-1}, \alpha_\mu, \alpha_\sigma|x_{1:t-1}) = \\ &p(\mu_t|\mu_{t-1}, \alpha\mu)p(\sigma_t|\sigma_{t-1}, \alpha_\sigma)p(\mu_{t-1}, \sigma_{t-1}, \alpha_\mu, \alpha_\sigma|x_{1:t-1}) \end{aligned}$$

$$(17)$$

... where $p(\mu_{t-1}, \sigma_{t-1}, \alpha_\mu, \alpha_\sigma | x_{1:t-1})$ is the posterior from the previous trial (hence the estimation is a Markov process).

Next we marginalise over the parameters from the previous trial:

$$p(\mu_{t-1}, \sigma_{t-1}, \alpha_\mu, \alpha_\sigma | x_{1:t-1}) = \int \mathrm{d}\mu_{t-1} \int \mathrm{d}\sigma_{t-1} \, \left[p(\mu_t, \mu_{t-1}, \sigma_t, \sigma_{t-1}, \alpha_\mu, \alpha_\sigma | x_{1:t-1}) \right] \tag{18}$$

... and finally incorporate the new data point from trial t

$$p(\mu_{t-1}, \sigma_{t-1}, \alpha_\mu, \alpha_\sigma | x_{1:t}) = \frac{p(x_t | \mu_t, \sigma_t) p(\mu_t, \sigma_t, \alpha_\mu, \alpha_\sigma | x_{1:t-1})}{\int \mathrm{d}\mu_t \int \mathrm{d}\sigma_t \int \mathrm{d}\alpha_\mu \int \mathrm{d}\alpha_\sigma \; p(x_t | \mu_t, \sigma_t) p(\mu_t, \sigma_t, \alpha_\mu, \alpha_\sigma)} \tag{19}$$

All integrals were performed using numerical grid integration.

At each trial, t the estimates of μ_t and σ_t used in fMRI modelling were the joint maximum likelihood values of $\{\mu_t, \sigma_t\}$, calculated after marginalising over α_μ and α_σ.
